# NRG4 suppresses breast cancer metastasis via ERBB4-YAP1-mediated down-regulation of MMPs

**DOI:** 10.1016/j.gendis.2025.101691

**Published:** 2025-05-16

**Authors:** Saijun Wang, Mingwei Guo, Lingyun Xu, Jiaming Xue, Shuai Chen, Ke Xu, Yan Zhou, Aihua Gu, Wei Gao, Jianwei Zhou, Yi Zhang, Liming Tang, Dongmei Wang

**Affiliations:** a Department of Gastrointestinal Surgery, Changzhou Medical Center, The Third Affiliated Hospital of Nanjing Medical University, The Affiliated Changzhou No.2 People's Hospital of Nanjing Medical University, Nanjing Medical University, Changzhou, Jiangsu 213000, China; b Shanghai Key Laboratory of Regulatory Biology, Institute of Biomedical Sciences, School of Life Sciences, East China Normal University, Shanghai 200241, China; c Department of Breast Surgery, The Affiliated Changzhou No. 2 People's Hospital of Nanjing Medical University, The Third Affiliated Hospital of Nanjing Medical University, Changzhou Medical Center, Nanjing Medical University, Changzhou, Jiangsu 213000, China; d Key Laboratory of Human Functional Genomics of Jiangsu Province, National Health Commission Key Laboratory of Antibody Techniques, School of Basic Medical Sciences, Nanjing Medical University, Nanjing, Jiangsu 211166, China; e State Key Laboratory of Reproductive Medicine, School of Public Health, Key Laboratory of Modern Toxicology of Ministry of Education, Center for Global Health, Nanjing Medical University, Nanjing, Jiangsu 211166, China; f Key Laboratory of Modern Toxicology of Ministry of Education, School of Public Health, Nanjing Medical University, Nanjing, Jiangsu 211166, China; g Shanghai Diabetes Institute, Shanghai Key Laboratory of Diabetes Mellitus, Shanghai Clinical Centre for Diabetes, Shanghai Sixth People's Hospital Affiliated to Shanghai Jiao Tong University School of Medicine, Shanghai 200233, China

**Keywords:** Breast cancer, Epithelial–mesenchymal transition, Matrix metalloproteinase alterations, Neuregulin 4, YAP

## Abstract

Obesity exacerbates breast cancer metastasis, yet the underlying mechanisms remain incompletely understood. Here, we identify neuregulin 4 (NRG4), a ligand of Erb-B2 receptor tyrosine kinase 4 (ERBB4), as a key regulator of metastasis, through the ERBB4-YAP1 signaling axis. Using MMTV-PyMT and 4T1 breast cancer models, we demonstrate that obesity accelerates metastasis, while NRG4, secreted by inguinal white adipose tissue (iWAT), inhibits cancer cell migration and epithelial–mesenchymal transition (EMT). Mechanistically, NRG4 activates ERBB4, producing a cleaved pERBB4 fragment that interacts with phosphorylated YAP1 (pYAP1), restricting its nuclear translocation. RNA sequencing revealed that NRG4 suppressed the transcription of Mmp9 and Mmp12, which encode matrix metalloproteinases critical for extracellular matrix remodeling and invasion. Co- immunoprecipitation and promoter assay confirmed that YAP1 bound to TEAD1 and activated MMP9/MMP12 transcription in the absence of NRG4. Importantly, recombinant NRG4 (rNRG4) reduced the growth and invasiveness of breast cancer organoids. These findings establish NRG4 as a metastasis suppressor in obesity-associated breast cancer by inhibiting the ERBB4-YAP1 pathway and down-regulating matrix metalloproteinases. Our study highlights the therapeutic potential of targeting NRG4-ERBB4 signaling to mitigate obesity-driven breast cancer progression.

## Introduction

Breast cancer is one of the most prevalent and lethal malignancies among women worldwide.^1^ Within its diverse subtypes, triple-negative breast cancer (TNBC) is particularly concerning due to its aggressive clinical course, early metastatic potential, and limited therapeutic options, which collectively result in poor prognoses and high mortality rates.^2^ Epithelial–mesenchymal transition (EMT) is a key driver of tumor metastasis, facilitating the acquisition of invasive and migratory phenotypes.^3^^,^^4^ While EMT's role in breast cancer progression has been extensively studied,^5^ the molecular mechanisms underpinning EMT in obesity-associated breast cancer remain poorly understood.

Obesity is a major risk factor for several cancers, including breast cancer.^6^ Epidemiological evidence reveals a strong correlation between obesity and breast cancer, with elevated body mass index linked to worse survival outcomes, diminished therapeutic responses, and lower rates of complete remission following neoadjuvant therapy.^7^, ^8^, ^9^, ^10^, ^11^, ^12^ Obesity-induced changes in adipose tissue function are known to influence the tumor microenvironment, promoting EMT and facilitating metastasis.^13^, ^14^, ^15^ Adipose tissues, including breast adipose tissue and inguinal white adipose tissue (iWAT), are abundant in adipocytes with high plasticity and secrete various proteins that regulate critical signaling pathways.^16^, ^17^, ^18^ These adipocyte-derived factors modulate tumor cell motility, invasion, and extracellular matrix remodeling by influencing cell–cell adhesion and matrix metalloproteinase activity.^19^, ^20^, ^21^ Despite these findings, the specific adipocyte-secreted proteins involved in promoting EMT and metastasis in the context of obesity remain unclear.

To address this gap, we performed a proteomic analysis of the secretome from C3H10T1/2 cells at different stages of adipocyte differentiation. This analysis identified neuregulin 4 (NRG4) as a candidate protein with significantly reduced expression in iWAT from obese models. NRG4, a member of the neurotrophic factor family, is primarily expressed in adipose tissue, liver, and other organs, where it regulates energy balance, glucose and lipid metabolism, and vascular homeostasis.^22^, ^23^, ^24^, ^25^, ^26^ Emerging evidence suggests that NRG4 exerts anti-inflammatory and anti-fibrotic effects, mitigates metabolic disorders, and modulates the tumor microenvironment.^23^^,^^26^, ^27^, ^28^ However, its role in breast cancer metastasis, particularly in obesity-associated contexts, has not been explored.

In this study, we systematically investigated the role of NRG4 in breast cancer metastasis, focusing on its mechanisms involving Erb-B2 receptor tyrosine kinase 4 (ERBB4) activation. Our findings reveal novel insights into the function of NRG4 as a metastasis suppressor and provide a foundation for potential therapeutic strategies targeting obesity-driven cancer progression.

## Materials and methods

### Clinical samples

In this study, we used a clinical tissue chip (80 TNBC cancer and 16 adjacent tissues) provided by Aifang Biological, China. All samples were voluntarily donated for scientific research with informed consent. Ethics approval and prior patient consent had been obtained from the Ethics Review Board of the Affiliated Changzhou No.2 People's Hospital of Nanjing Medical University, Changzhou Medical Center, Nanjing Medical University ([2024]KY101-01).

### Analysis of public data

RNA sequencing data and corresponding clinical information of The Cancer Genome Atlas (TCGA)-breast cancer cohort were downloaded from the TCGA database (https://portal.gdc.cancer.gov/). Expression correlation analysis was performed by GEPIA2 (http://gepia2.cancer-pku.cn) or UALCAN (https://ualcan.path.uab.edu/) online web tools. Survival analysis was performed with Kaplan–Meier Plotter (https://kmplot.com/) database web tools.^29^ Clinical data were obtained from TCGA using Kaplan–Meier Plotter, and analyzed using the R packages survival and survminer. Protein interaction analysis was performed by STRING (https://cn.string-db.org/).

### Animal experiments

All mice were housed in a temperature-controlled room at 22 °C with a 12 h/12 h light/dark cycle and provided with free access to food and water. All animal studies were carried out following the guidelines approved by the Ethics Committee of Animal Experiments of Nanjing Medical University (Approval No. IACUC-2308039).

Eight-week-old mouse mammary tumor virus-polyomavirus middle T-antigen (MMTV-PyMT) FVB mice (Gempharmatech, China) and BalB/C female mice were used in the study. For high-fat diet (HFD)-induced obesity studies, 8-week-old MMTV-PyMT FVB mice were fed with an HFD (ResearchDiet, D12492) for the indicated times and monitored for changes in body weight. Normal-chow diet (NCD)-fed mice were used as controls. Mice were subsequently sacrificed, and tissues were dissected for further analysis. In addition, 8-week-old BalB/C mice were fed with an HFD for 7-9 weeks and monitored for changes in body weight; then the mice were intratumorally injected with 5 × 10^5^ 4T1 cells into the fourth mammary gland, and tumor volume was monitored every three days for 3–4 weeks. In the end, the mice were sacrificed, and the tissues were dissected for further analysis.

To analyze the function of NRG4 in cancer metastasis, 8-week-old MMTV-PyMT FVB female mice were subjected to injection with AAV-NRG4 in iWAT and then fed with an HFD for 7–9 weeks, and body weight was monitored every week. Mice were subsequently sacrificed, and tissues were dissected for further analysis. In addition, 8-week-old female BalB/C mice were fed an HFD for 7–9 weeks and then intratumorally injected with breast cancer cells into the fourth mammary gland. One week later, 100 ng/iWAT rNRG4 was administered to the mice, and the tumor volume was monitored every three days for 3–4 weeks. Eight-week-old nude mice were injected with ERBB4 overexpressed MDA-MB-231 cells via the tail vein, and subsequently sacrificed, and tissues were dissected for further analysis.

### Cell culture

4T1, MDA-MB-231, MDA-MB-453, MCF10A, MCF7, T47D, BT549, MDA-MB-468, and HEK293T cells were obtained from the American Type Culture Collection (Manassas, VA, USA). 4T1, MCF7, T47D, BT549, and MDA-MB-468 cells were cultured in RPMI 1640 medium, while MDA-MB-231, MDA-MB-453, and HEK293T cells were cultured in high-glucose Dulbecco's modified Eagle medium (DMEM). MCF10A cells were cultured in DMEM supplemented with 100 ng/mL cholera toxin, 10 μg/ml insulin, 20 ng/ml EGF, 0.5 μg/ml Hydrocortisone. All media were supplemented with 10% fetal bovine serum (FBS), 100 U/mL penicillin, and 100 mg/mL streptomycin. The cells were maintained in a humidified incubator at 37 °C with 5% CO_2_, and the medium was refreshed every 2–3 days. Upon reaching 90% confluence, the medium was aspirated, and trypsin was added for cell detachment and subsequent subculture.

### Co-culture assay

Equal amounts of freshly isolated tissues were generated from the same mouse and cultured in 24-well plates containing DMEM/F12 supplemented with 10% FBS, 100 U/mL penicillin, and 100 mg/mL streptomycin for 24 h. Following this, 4T1 or MDA-MB-231 cells were seeded into the upper chamber of a transwell apparatus. 16 h later, the cells were collected for gene expression and cell migration analysis.

### Conditioned medium preparation

Preadipocyte or differentiated immortalized beige adipocytes or primary adipocytes were cultured under normal conditions. Subsequently, the cells were switched to fresh DMEM without FBS for 24 h. The conditioned medium from mature adipocytes or preadipocytes was then collected. For the preparation of conditioned medium from tissues, equal amounts of fresh tissue were obtained from mice, minced, and washed with precooled PBS solution three times. The tissues were then cultured in serum-free fresh DMEM without FBS for 24 h. Subsequently, the conditioned medium was collected for further analysis.

### Recombinant NRG4 (rNRG4) protein purification

The mouse NRG4 (1–61, E47Q) recombinant protein was expressed and purified using a pET expression system (Novagen) according to a previous report.^30^

### Plasmid construction

The CDS regions of mouse or human ERBB4 and Yap5SA (5 serine sites were mutated to alanine) were cloned and inserted into the pCDH-CMV-MCS-EF1-Puro vector as previously described.^31^ shERBB4 was cloned and inserted into pLKO.1. Lentiviruses were then packaged in 293T cells. The promoter sequences of Mmp9 and Mmp12 spanning from −2000 bp to +500 bp were cloned and inserted into pGL3basic vectors. The primers used to construct these plasmids are listed in Table S1.

### Transwell assay

Cells from different experimental groups were harvested, trypsinized for digestion, centrifuged, and resuspended in culture medium. Cell counting was conducted, and the cell density was adjusted to 1 × 10^6^ cells/mL. Subsequently, 200 μL of the cell suspension without serum was seeded into the upper chamber of a transwell apparatus. The lower chamber was filled with 600 μL of DMEM containing 10% FBS. 16 h later, the cells from the upper chamber were fixed with 4% paraformaldehyde for 15 min. HE staining was then performed according to the manufacturer's instructions (Beyotime). Three to five random fields of view were selected for cell counting under an inverted microscope.

### Beige adipocyte cell culture and differentiation

Immortalized beige adipocytes and primary iWAT stromal vascular fractions were cultured in DMEM (Gibco) supplemented with 20% FBS (Gibco, 10270) and 1% streptomycin and penicillin (Gibco, 15070063). For beige adipocyte differentiation, immortalized beige preadipocytes were induced in differentiation medium containing 5 μg/mL insulin (Sigma, I9278), 0.5 mM IBMX (Sigma, I5879), 1 μM dexamethasone (Sigma, D4902), and 1 μM rosiglitazone (Sigma, R2408) for 2 days. Subsequently, the cells were cultured in maintenance medium supplemented with 1 μM rosiglitazone and 5 μg/mL insulin. Primary iWAT stromal vascular fractions were induced to differentiate into beige adipocytes with an adipogenic cocktail (6 μg/mL insulin, 0.5 mM IBMX, 1 μM dexamethasone, 50 nM T3, and 1 μM rosiglitazone) in DMEM containing 10% FBS and 1% penicillin/streptomycin for 2 days. The cells were then maintained in maintenance medium containing 50 nM T3, 1 μM rosiglitazone, and 6 μg/mL insulin. The maintenance medium was refreshed every two days, and mature adipocytes were collected on the 7th day. All cells were cultured at 37 °C in a humidified incubator with 5% CO_2_.

### RNA extraction and quantitative PCR

Total RNA was extracted from cells or tissues using TRIzol reagent following the manufacturer's protocol (TaKaRa). Then, 1 μg of RNA was reverse-transcribed to complementary DNA (cDNA) using PrimeScriptTM RT Master Mix (TaKaRa). Quantitative reverse-transcription PCR (qPCR) was performed using HieffTM qPCR SYBR Green Master Mix (Low Rox Plus) (Yeasen). Gapdh was utilized as the reference gene for data normalization. The sequences of primers used are listed in Table S1.

### RNA sequencing

For RNA sequencing analysis, RNA quality was evaluated using a standard sensitivity RNA analysis kit. High-quality RNA samples were utilized to construct libraries, and a fragment analyzer was used for RNA sequencing. Briefly, RNA libraries were prepared using the TruSeq RNA LT Sample Prep Kit v2 following the manufacturer's protocol. The processed RNA sequencing data were obtained on the BGISEQ platform. Read counts were normalized using trimmed mean of M-values (TMM) normalization, and counts per million were calculated to generate a matrix of normalized expression values. Differential gene expression was determined using a significance threshold of *p* < 0.05 and |fold change| ≥ 2. For the Kyoto Encyclopedia of Genes and Genomes (KEGG) enrichment analysis, clusterProfiler (v3.12.0) was utilized, with significance set at a *p*-value <0.05 for identifying enriched pathways.

### NRG4 content

Measurements of NRG4 level were conducted by homogenizing adipose tissues, mammary tissues, tumors, or cells in ice-cold phosphate-buffered saline solution, followed by centrifugation at 12,000 rpm and 4 °C for 10 min. NRG4 levels were quantified using an NRG4 ELISA kit according to the manufacturer's instructions (Zeye, Shanghai).

### Immunoblotting

Cells and tissues were lysed using a RIPA lysis buffer (Beyotime, Shanghai) supplemented with phenylmethylsulfonyl fluoride (Beyotime, Shanghai) and protease inhibitors (Beyotime, Shanghai). Total protein lysates were then denatured with loading sample buffer containing 10% SDS-PAGE. The protein samples were separated by SDS-PAGE and transferred onto nitrocellulose membranes. Nitrocellulose membrane blots were blocked with 10% skim milk at room temperature for 1 h, followed by washing in Tris-buffered saline with Tween 20. Subsequently, the membranes were incubated at 4 °C overnight with primary antibodies. The antibodies used are listed in Table S2.

### Hematoxylin and eosin (HE) staining and immunohistochemistry staining

Breast cancer and adjacent tissues were fixed in 10% formalin for 12–24 h, embedded in paraffin, and sectioned. The sections were then stained with hematoxylin and eosin (Beyotime) according to the manufacturer's instructions. Images were captured using an optical microscope (Nikon) with a 20 × objective. Immunohistochemistry staining of NRG4 and ERBB4 was conducted according to our previous report.^31^ The antibodies used are listed in Table S2. Images of the immunostained sections were captured using an optical microscope (Nikon).

### Dual-luciferase reporter assay

HEK293T cells were seeded and cultured in 24-well plates for 24 h. Next, the cells were transfected with either the pGL4.17 reporter vector containing Mmp9 or Mmp12 along with the SV40 and pCDH-YAP1 plasmids. The relative luciferase reporter activity was assessed using the double-luciferase reporter assay kit according to the manufacturer's instructions outlined in the manuscript.

### Liquid chromatography-mass spectrometry (LC/MS) analysis

The silver staining procedure was performed using a Fast silver stain kit (Beyotime) following the manufacturer's instructions. The specific bands or conditioned media were analyzed by LC/MS, where liquid chromatography is the U3000 (Thermo Fisher) and tandem mass spectrometry is Timstof Pro-2 (Bruker). Protein identification and quantification were carried out using PEAKS software (Bioinformatics Solutions, Inc.). Proteins meeting the criteria of an area ratio (sense/antisense fold change) > 2 and unique peptides > 2 were considered significant. The datasets generated during this study are available at integrated proteome resources iProX with the dataset identifier IPX0008974000 (https://www.iprox.cn/page/PSV023.html; url = 1724207801199cHd0, code: BS3t). ERBB4 binding proteins in 4T1 cells from LC/MS assay are listed in Table S3.

### Immunofluorescence

The cells were fixed in 10% formalin, followed by blocking and staining in antibody diluent containing 10% goat serum. Subsequently, the cells were incubated at 4 °C overnight with rabbit anti-Yap1 (S127) or mouse anti-ERBB4 antibodies. Afterward, the cells were treated with the corresponding secondary antibodies, and the antibodies used are listed in Table S2. Finally, the cells were mounted with 4′,6-diamidino-2-phenylindole and imaged using fluorescence microscopy.

### Organoids formation, culture, and subculture

Patient-derived breast cancer tissues were thoroughly washed 4 to 6 times with organoid washing buffer (LSTO00200201, Shanghai Lisheng Biotech, China) and then cut into approximately 1 mm diameter pieces using surgical scissors, without collagenase, trypsin, or dispase. The tumor pieces were then resuspended in breast cancer organoid medium (LSTO00200402, Shanghai Lisheng Biotech, China). The organoids were maintained at 37 °C with 5% CO_2_, and the medium was changed semi-weekly. Once the diameter of the organoids reached 2 mm (approximately 2 weeks), they were collected and passaged for further treatment. The ethics approval number is [2024]KY101-01.

### Statistical analysis

GraphPad Prism 8.0 (GraphPad, San Diego, CA, USA) was utilized for data analysis. Detailed statistical information can be found in the figure legends. Student's *t*-test was used to compare two groups, while two-way ANOVA with Bonferroni's multiple comparisons test was used for comparisons involving multiple factors. The results were presented as mean ± standard deviation, and statistical significance was set at *p* < 0.05. Significance levels are denoted as follows: ∗*p* < 0.05, ∗∗*p* < 0.01, ∗∗∗*p* < 0.001, and n.s. (no significance).

## Results

### Obesity promotes breast cancer metastasis

**Figure 1:**
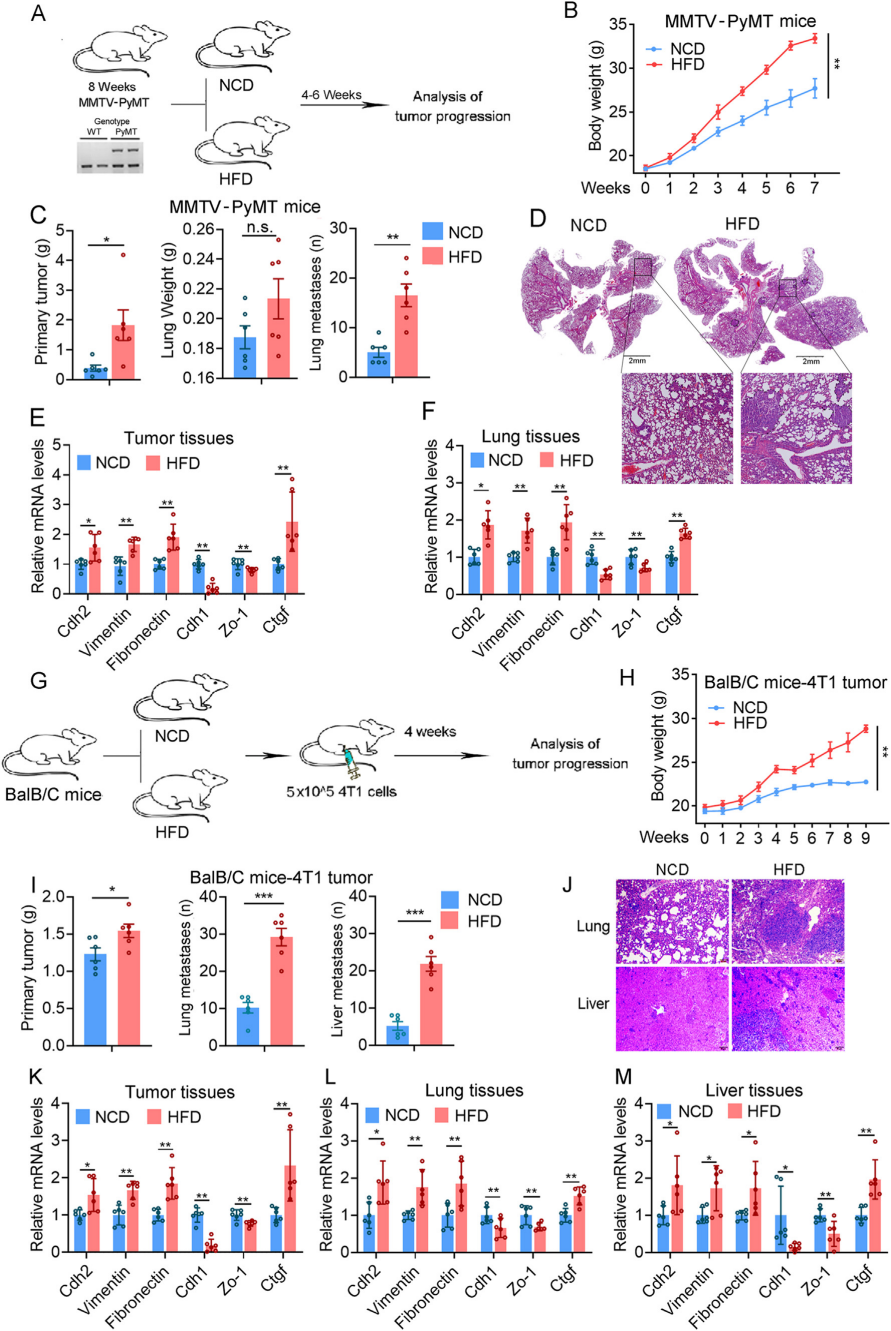
Obesity promotes breast cancer metastasis. **(A)** The HFD-induced MMTV-PyMT mouse model. **(B)** Body weight changes in HFD-induced MMTV-PyMT mice (*n* = 6 for each group). **(C)** Obesity increases the weight of primary mammary tumors and the number of lung metastases of MMTV-PyMT mice. **(D**–**F)** Representative microscopic images of lung tissues (D), relative mRNA levels (normalized to Gapdh) of the EMT-related genes and Ctgf gene in the 4th pair of mammary tumors (E) and metastatic lung tissues (F) of MMTV-PyMT mice (*n* = 6) fed with a HFD. **(G)** Model of the HFD-induced 4T1 breast intra-tumoral model. **(H)** Body weight changes in the HFD-induced 4T1 breast intra-tumoral model (*n* = 6 for each group). **(I)** Obesity increases primary tumor weight and the number of metastases in lung and liver tissues after the inoculation of 4T1 cells in mice. **(J**–**M)** Representative microscopic images of lung tissues (J), relative mRNA levels (normalized to Gapdh) of the EMT-related genes and Ctgf gene in the primary tumors (K), lung tissues (L), and liver tissues (M) of the 4T1 tumor-bearing mice (*n* = 6) fed with a high-fat diet. The data were presented as mean ± standard deviation. ∗*p* < 0.05, ∗∗*p* < 0.01, and ∗∗∗*p* < 0.001. n.s., no significance.

To test the effect of obesity on breast cancer metastasis, we utilized female transgenic mice carrying MMTV-PyMT, which spontaneously develop palpable mammary tumors resembling human breast cancers.^32^ These mice were fed with an HFD to induce obesity (Fig. 1A). Unlike their counterparts fed with an NCD, HFD-fed mice exhibited significant weight gain (Fig. 1B) with a substantial increase in lung metastasis (Fig. 1C and D). Furthermore, HFD nearly doubled the expression of mesenchymal-related genes N-cadherin (Cdh2), vimentin, and fibronectin, while significantly reducing the expression of epithelial-related genes E-cadherin (Cdh1) and zonula occludens 1 (Zo-1) by approximately 50% in both primary tumors and lung tissues (Fig. 1E and F). Additionally, HFD promoted the expression of the angiogenesis-related gene connective tissue growth factor (Ctgf) (Fig. 1E and F). The invasive nature of MMTV-PyMT tumors prompts us to hypothesize that obesity has a pivotal impact on breast cancer metastasis. We also investigated the effect of HFD on the metastasis of breast cancer by establishing a mouse model through mammary injection of the mouse TNBC cell line 4T1 following HFD feeding^33^ (Fig. 1G and H). Consistently, we detected significant lung and liver metastases in HFD-fed mice (Fig. 1I and J). HFD nearly doubled the expression of mesenchymal genes (Cdh2, Vimentin, and Fibronectin) and Ctgf while markedly reducing the expression of epithelial genes (Cdh1 and Zo-1) by approximately 50% (Fig. 1K–M). Protein levels of Vimentin (Fig. S1) of different tissues from the breast cancer metastatic mice were also increased in the HFD group. These results underscore the role of obesity in promoting cancer metastasis of both invasive breast cancer and TNBC.

### NRG4, secreted by iWAT tissues, possesses the potential to inhibit the invasion and metastasis of breast cancer

**Figure 2:**
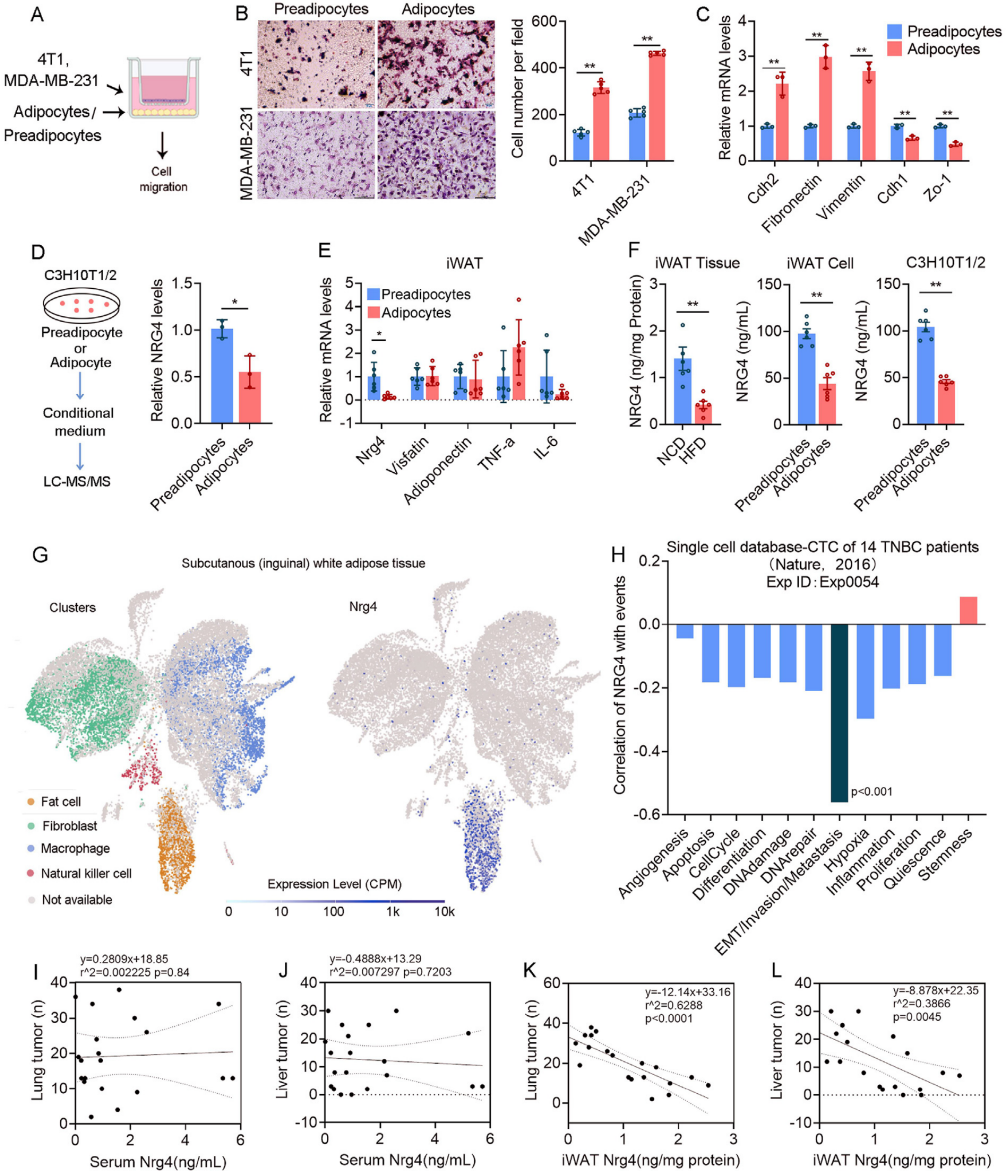
NRG4, secreted by iWAT, possesses the potential to inhibit the invasion and metastasis of breast cancer. **(A**–**C)** Coculture of pre- or post-differentiated iWAT cells with breast cancer cells (A), followed by transwell (B) and qPCR experiments (C) for assessing cell migration. **(D)** Protein mass spectrometry analysis of conditioned media from pre- or post-differentiated C3H10T1/2 cells was carried out to identify commonly secreted proteins, such as NRG4. **(E)** qPCR analysis of secreted protein expression in the primary iWAT cells isolated from HFD-fed- and NCD-fed mice. **(F)** ELISA was performed to detect the NRG4 content in iWAT from mice with different metabolic statuses and the culture media from primary iWAT cells and C3H10T1/2 cells at various stages of differentiation. **(G)** Single-cell transcriptomic data of subcutaneous adipose tissues showed that NRG4 was expressed in adipocytes. **(H)** Single-cell transcriptomic data from circulating tumor cells (CTCs) of 14 TNBC patients indicated that NRG4 had the strongest negative correlation with tumor cell invasion and metastasis. **(I**–**L)** The correlation between the expression of NRG4 in serum (I, J) or adipose tissue (K, L) and the number of tumor metastases in the lung and liver in 4T1 tumor-bearing mice. The data were presented as mean ± standard deviation. ∗*p* < 0.05 and ∗∗*p* < 0.01.

As both mammary and iWAT tissues are located near breast tumor cells, there is currently no evidence to determine which tissue or cell type plays a critical role in breast tumor cell migration. An *in vitro* co-culture experiment was used to examine the impact of adjacent tissues on obesity-induced breast cancer cell migration. Results showed that mammary tissue from obese individuals did not significantly affect breast cancer cell migration (Fig. S2A), while iWAT from obese individuals significantly promoted breast cancer cell migration (Fig. S2B). This suggests that iWAT, as a highly plastic secretory organ adjacent to breast tissue, plays a crucial role in promoting breast cancer cell migration. Given that adipocytes are prominent constituents of adipose tissue, we conducted a detailed investigation into the impact of pre- and post-differentiated primary adipocytes of iWAT on the migration of breast cancer cells. Our findings indicate that mature adipocytes notably enhance breast cancer cell migration (Fig. 2A–C). To identify these factors that influence cell migration, we obtained cell culture media from C3H10T1/2 cells before and after adipocyte differentiation induction. Mass spectrometry analysis indicated a significant decrease in NRG4 among the 237 proteins present in culture media, indicating that NRG4 expression is higher in preadipocytes compared with adipocytes (Fig. S3A, B; Fig. 2D). Further investigation into the mRNA levels of Nrg4 and other adipocyte-secreted proteins revealed a notable decrease in Nrg4 mRNA levels specifically within adipocytes, as opposed to their precursor cells (Fig. 2E). Analysis of NRG4 protein levels in iWAT tissue and adipocytes demonstrated a significant decrease in NRG4 expression in the mature, well-differentiated subcutaneous adipocytes of obese mice (Fig. 2F).

Subsequent reevaluation of single-cell RNA sequencing data from subcutaneous adipose tissue (SSA: ERP135494) indicated that adipocytes were the primary location of NRG4 distribution (Fig. 2G). A thorough analysis of single-cell RNA sequencing data (Exp ID: Exp0054) obtained from circulating tumor cells of patients with breast cancer demonstrated a notable inverse association between NRG4 expression levels and EMT, cellular invasion, or tumor metastasis (Fig. 2H). Subsequent investigation into the relationship between NRG4 and metastatic tumor burden indicated a lack of significant correlation between serum NRG4 levels from 4T1 tumor-bearing mice and tumor metastasis (Fig. 2I and J). Conversely, a significant negative correlation was observed between NRG4 levels in the subcutaneous adipose tissue of 4T1 tumor-bearing mice and tumor metastasis (Fig. 2K and L). These results strongly suggest that NRG4, secreted by subcutaneous adipocytes, may have potential as a tumor suppressor through paracrine signaling in regulating the metastatic potential of breast cancer. .

### NRG4 inhibits the cell migration and metastasis of breast cancer

**Figure 3:**
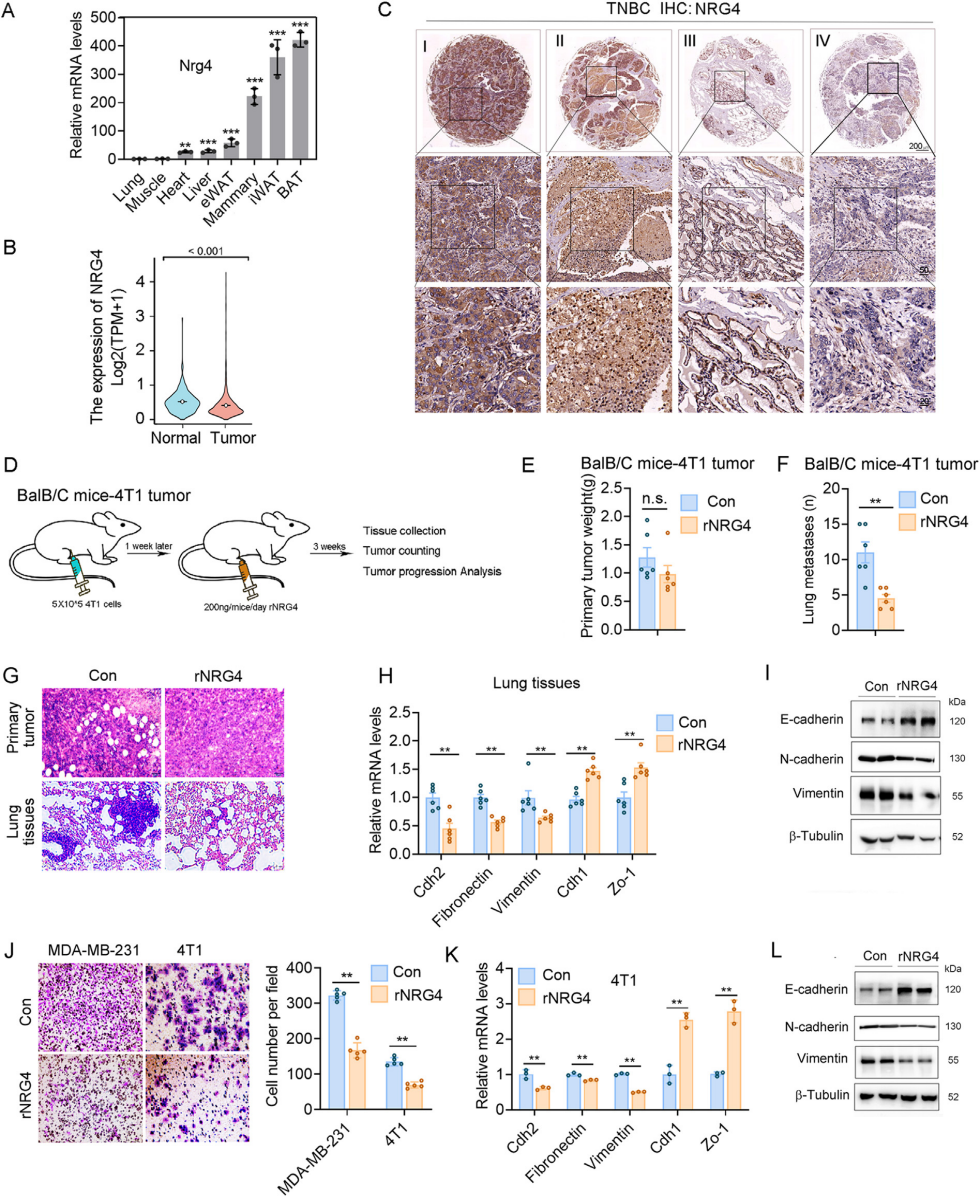
NRG4 inhibits the cell migration and metastasis of breast cancer. **(A)** qPCR analysis of NRG4 expression in different tissues of mice fed a chow diet revealed high expression of NRG4 in adipose tissue. **(B)** TCGA data revealed a significant decrease in NRG4 expression in invasive breast cancer tissue compared with adjacent tissue. **(C)** Immunohistochemistry analysis demonstrated a significant decrease in NRG4 expression in late-stage breast cancer tissue. **(D)** Schematic diagram of the model of obese mice bearing breast tumors treated with rNRG4. **(E, F)** rNRG4 treatment did not affect the weight of primary tumors (E) but inhibited lung metastases of 4T1 tumor-bearing mice (*n* = 6) (F). **(G)** HE staining was used to detect the morphology of primary tumors and lung tissues. **(H)** qPCR analysis showed that the expression of migration-related genes in primary tumor cells was inhibited by rNRG4 (*n* = 6). **(I)** Immunoblotting analysis showed that the protein expression of migration-related genes in lung tissue was inhibited by rNRG4 after tumor metastasis. **(J)** Transwell experiments demonstrated the inhibition of cell migration by rNRG4. **(K, L)** qPCR and Immunoblotting analysis showed the expression of migration-related genes inhibited by rNRG4 in breast cancer cells. The data were presented as mean ± standard deviation. ∗*p* < 0.05, ∗∗*p* < 0.01, and ∗∗∗*p* < 0.001. n.s., no significance.

To further explore the role of NRG4 in the invasion and metastasis of breast cancer, we initially assessed its expression in various tissues of mice fed with a NCD and observed a notable increase in adipose tissues (Fig. 3A). At the cellular level, NRG4 expression was found to be higher in preadipocytes (Fig. 2D), its secretion as an adipokine predominantly occurs in preadipocyte-rich iWAT and BAT (Fig. 3A). Analysis of TCGA database revealed a significant decrease in NRG4 expression in tumor tissues compared with adjacent tissues in cases of invasive breast cancer (Fig. 3B). Utilizing a breast cancer tissue chip, we investigated the association between NRG4 expression and cancer progression. Our findings revealed a gradual decrease in NRG4 expression in breast cancers as the disease advanced (Fig. 3C). Collectively, these findings suggest that NRG4 functions as a potent breast tumor suppressor.

To further explore the impact of NRG4 in adipose tissue on tumor metastasis, 4T1 breast cancer-bearing mice were administered recombinant NRG4 (rNRG4) targeted at adipose tissue (Fig. 3D–I). While rNRG4 treatment had minimal effects on the weight of primary breast cancer tumors (Fig. 3E), it notably hindered the development of metastatic lung tumors (Fig. 3F and G). Further examination of the EMT status in lung tissue indicated that rNRG4 effectively suppressed the expression of genes and proteins linked to EMT (Fig. 3H and I; Fig. S4A). Moreover, our study demonstrated that the administration of AAV-Nrg4 into iWAT resulted in a reduction of breast cancer cell metastasis to the lungs in MMTV-PyMT model mice (Fig. S4B, C). Additionally, our investigation into the impact of rNRG4 on breast cancer cell migration indicated a significant inhibition of cell migration and EMT (Fig. 3J–L) without affecting cell proliferation (Fig. S4D). Therefore, NRG4 significantly impedes the metastasis and migration of breast cancer tumors.

### NRG4 mediated the inhibition of breast cancer metastasis through ERBB4 phosphorylation

**Figure 4:**
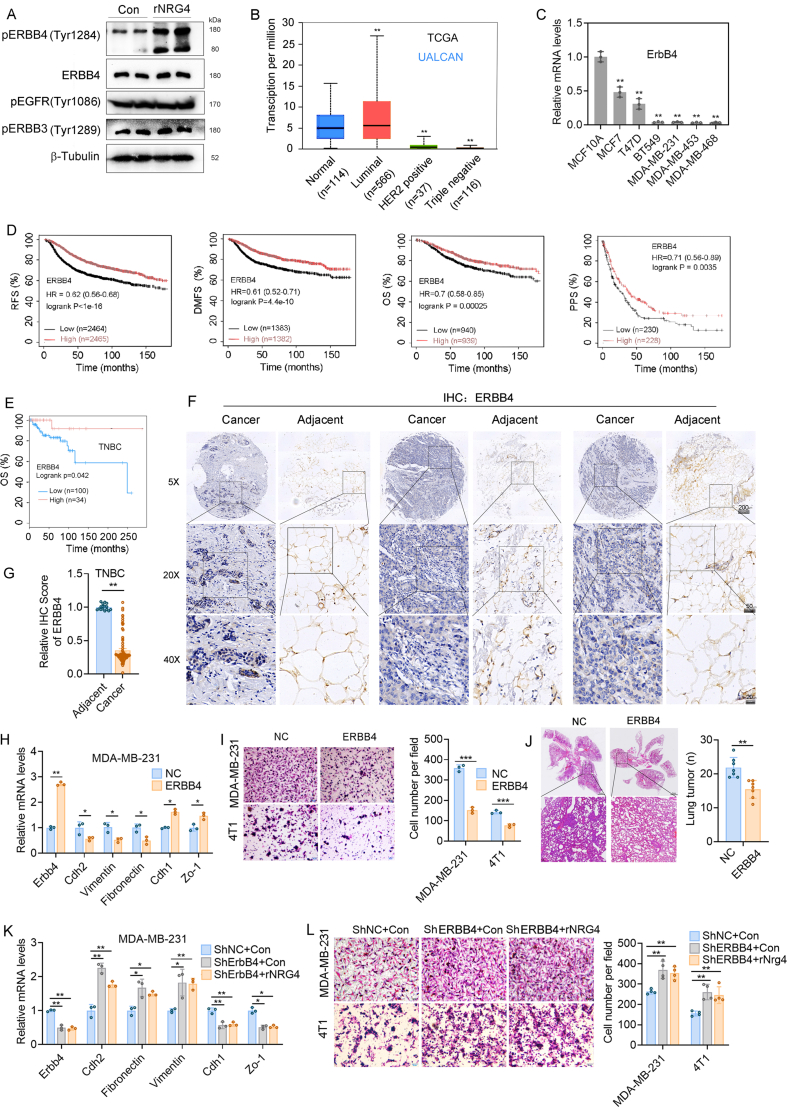
NRG4 inhibits breast cancer metastasis via ERBB4 phosphorylation. **(A)** Immunoblotting experiments indicated that rNRG4 significantly enhanced ERBB4 phosphorylation and cleavage in MDA-MB-231 cells. **(B)** Analysis of ERBB4 expression across different subtypes of breast cancer using the TCGA database. **(C)** qPCR experiments revealed the expression of ErbB4 in breast epithelial cells. **(D)** Kaplan–Meier survival analysis based on TCGA data demonstrated a significant positive correlation between ERBB4 expression and patient survival. **(E)** The survival curve illustrating the correlation between ERBB4 expression and survival among breast cancer patients. **(F, G)** Immunohistochemistry experiments revealed significantly lower ERBB4 expression in clinical breast cancer tissue than in adjacent tissue. **(H)** qPCR was used to evaluate the impact of ERBB4 overexpression on EMT. **(I)** Transwell experiments demonstrated the influence of ERBB4 on cell migration. **(J)** HE staining (left) and tumor number of lung tissues of nude mice injected with ERBB4-overexpressed MDA-MB-231 cells via the tail vein. **(K)** qPCR experiments demonstrated the effect of NRG4 on EMT induced by ERBB4 gene knockdown. **(L)** Transwell experiments revealed the effect of NRG4 on cell migration induced by ERBB4 gene knockdown. The data were presented as mean ± standard deviation. ∗*p* < 0.05, ∗∗*p* < 0.01, and ∗∗∗*p* < 0.001.

Given the established role of NRG4 as a ligand for ERBBs, our study sought to elucidate the mechanisms underlying NRG4-mediated inhibition of breast cancer cell migration by evaluating the phosphorylation status of canonical ERBB family receptor proteins post-NRG4 treatment. Our results demonstrated a marked enhancement in ERBB4 phosphorylation in breast cancer cells upon NRG4 exposure in MDA-MB-231 cells (Fig. 4A). To further investigate the clinical relevance of ERBB4 in breast cancer, we conducted an analysis of ERBB4 expression levels in various subtypes of breast cancer patients using data from the TCGA database. Subsequently, elevated ERBB4 expression was observed in luminal breast cancer patients, whereas HER2-positive and TNBC patients exhibited significantly lower levels of ERBB4 expression (Fig. 4B; Fig. S5A). Moreover, relative ERBB4 expression in breast cancer cells was notably diminished compared with normal mammary epithelial cells (Fig. 4C). Additionally, the relative ERBB4 expression in primary tumors and lung metastases of HFD-fed mice was lower than that in tissues of NCD-fed mice (Fig. S5B). Nonetheless, a significant increase in ERBB4 expression levels was observed in the tissues of mice treated with rNRG4 (Fig. S5C). The study examined the clinical relevance of ERBB4 expression levels in relation to the survival outcomes of breast cancer patients, utilizing data from the TCGA database. The findings indicated a positive association between elevated ERBB4 expression and increased survival rates in breast cancer patients (Fig. 4D). Additionally, an analysis of ERBB4 expression levels in TNBC patients demonstrated a positive correlation with patient survival (Fig. 4E). Following this, ERBB4 expression was examined in tumor and adjacent tissues through the analysis of 16 adjacent tissues and 80 TNBC tumor tissues. The study revealed a notable decrease in ERBB4 expression in tumor tissues compared with adjacent tissues, including adipose tissues (Fig. 4F and G).

To delve deeper into the function of ERBB4 in breast cancer cell migration, ERBB4-overexpressing MDA-MB-231 and 4T1 stable clones were generated. The findings demonstrated that ERBB4 played a significant role in inhibiting EMT, cell migration, and MDA-MB-231 lung metastasis (Fig. 4H–J). Significantly, following the down-regulation of ERBB4 in MDA-MB-231 and 4T1 cells, an observed enhancement in EMT and cell migration was noted. Previous data showed that rNRG4 inhibited EMT-related gene and protein expression and metastasis (Fig. 3F–L), while knockdown of ERBB4 reversed the rNRG4-induced inhibition of EMT (Fig. 4K and L) without significantly affecting cell proliferation (Fig. S5D). This implies a crucial involvement of ERBB4 in facilitating the inhibitory actions of rNRG4 on cancer metastasis.

### NRG4 promotes the interaction between pERBB4 and pYAP1 to hinder the nuclear translocation of YAP1

**Figure 5:**
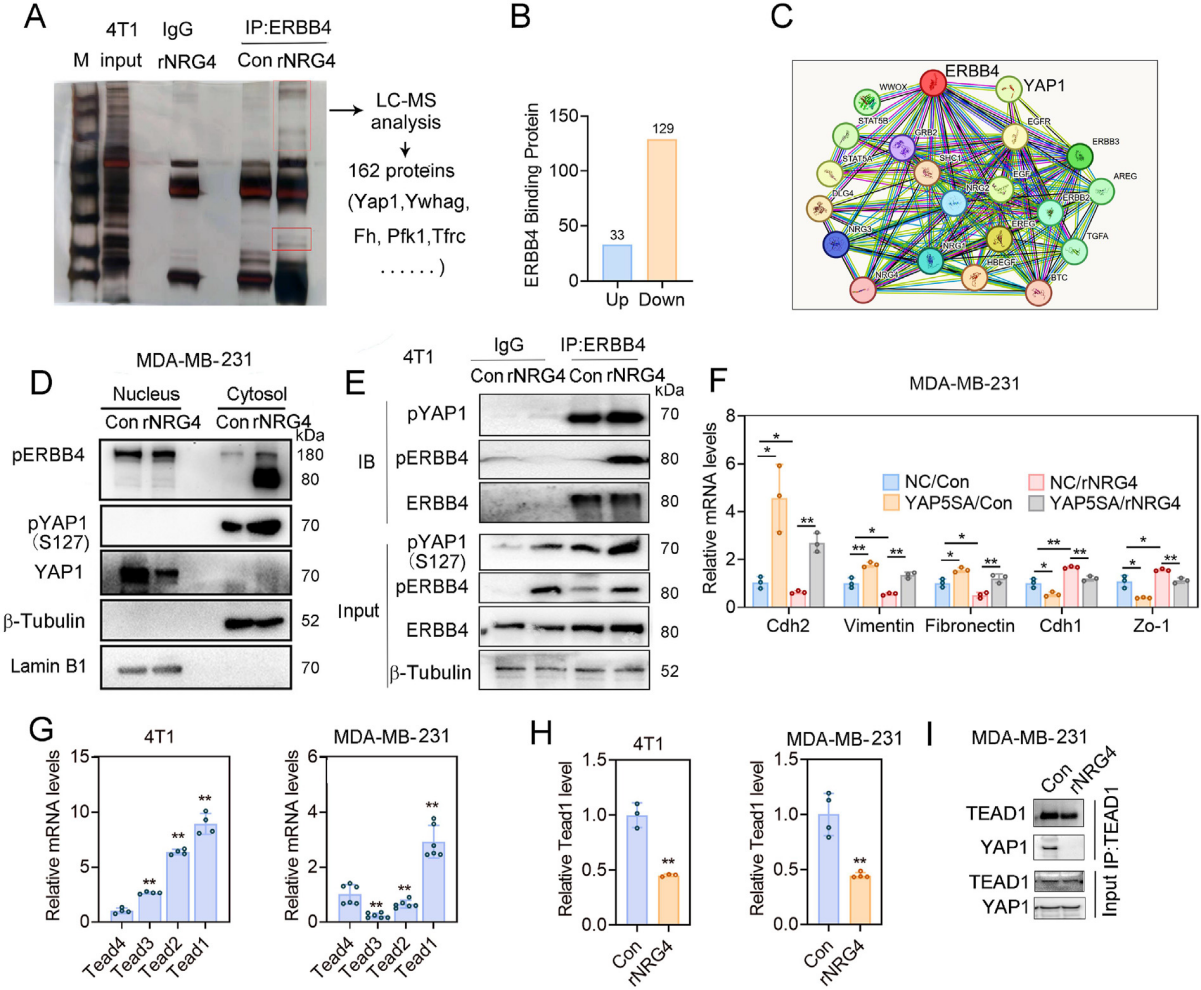
NRG4 facilitates the interaction between pERBB4 and pYAP1 to hinder the nuclear translocation of YAP1. **(A)** Co-IP was used to enrich for ERBB4-bound proteins, followed by silver staining of differential bands and protein mass spectrometry for identifying 162 binding proteins in 4T1 cells. **(B)** The number of ERBB4 binding proteins in LC/MS assays upon rNRG4 treatment for 24 h. **(C)** STRING analysis revealed the interaction between ERBB4 and YAP1. **(D)** Western blotting analysis after nuclear-cytoplasmic fractionation revealed increased pERBB4 and pYAP1 in the cytoplasm and reduced YAP1 expression in the nucleus after rNRG4 treatment. **(E)** Co-IP of ERBB4 in the cytoplasm of 4T1 cells treated with rNRG4, followed by immunoblotting. **(F)** qPCR analysis of the mRNA expression levels of EMT-related genes in MDA-MB-231 cells overexpressing YAP5SA in the presence of NRG4. **(G)** qPCR analysis of the expression levels of Tead subunits in 4T1 cells and MDA-MB-231 cells. **(H)** qPCR analysis of the mRNA expression levels of Tead1 in 4T1 cells and MDA-MB-231 cells treated with 100 ng/mL rNRG4. **(I)** Co-IP results demonstrated the decrease of TEAD1 and YAP1 binding by rNRG4 treatment. The data were presented as mean ± standard deviation. ∗*p* < 0.05 and ∗∗*p* < 0.01.

To further understand the role of ERBB4 in regulating cell migration, co-immunoprecipitation assays were conducted using an ERBB4 antibody in 4T1 cells, followed by silver staining and LC/MS analysis of specific protein bands. We identified 162 ERBB4 binding proteins, including Yap1, Ywhag, Fh, Tfrc, and Pfk1 (Fig. 5A; Table S3). Among these, 33 were up-regulated and 129 were down-regulated by rNRG4 (Fig. 5B). In addition, our examination of the STRING database indicated a connection between ERBB4 and YAP1 (Fig. 5C). YAP1 is a transcriptional coactivator that can activate the transcription enhancer associated domain transcription factor after the inactivation of Hippo signaling pathway, thereby regulating biological processes such as proliferation, survival, and differentiation.

Subsequently, we explored the interaction between YAP1 and ERBB4 in various compartments of MDA-MB-231 cells. Our findings demonstrated that rNRG4 treatment increased the expression of pERBB4 and pYAP1 in the cytosol while decreasing the YAP1 level in the nucleus (Fig. 5D). Moreover, co-immunoprecipitation of ERBB4 in the cytosolic compartment indicated that rNRG4 enhanced the interaction between pYAP1 and pERBB4 (Fig. 5E), and immunofluorescence analysis of pYAP1 and ERBB4 in 4T1 cells demonstrated that ERBB4 promoted the colocalization of them (Fig. S5E), suggesting that the interaction between pYAP1 and pERBB4 in the cytosol may inhibit pYAP1 nuclear translocation. To further confirm the role of pYAP1 in breast cancer cell EMT, YAP5SA, a mutant with five serine residues replaced by alanine, which can promote nuclear translocation, was overexpressed, effectively reversing the inhibitory effect of rNRG4 on EMT (Fig. 5F). Previous studies have suggested that YAP1 acts as a coactivator for various transcription factors, including transcriptional enhanced associate domains (TEADs). In this study, we examined the expression levels of TEADs in NRG4-treated breast cancer cells and found that TEAD1 displayed the highest expression, indicating a potential responsiveness to rNRG4 treatment (Fig. 5G and H). Furthermore, we found that rNRG4 could inhibit the binding of TEAD1 and YAP1 (Fig. 5I). Overall, these results support the conclusion that NRG4 suppresses EMT by facilitating the interaction between pERBB4 and pYAP1, thereby suppressing the nuclear translocation of YAP1.

### NRG4 suppresses breast cancer metastasis by inhibiting the transcription of MMPs

**Figure 6:**
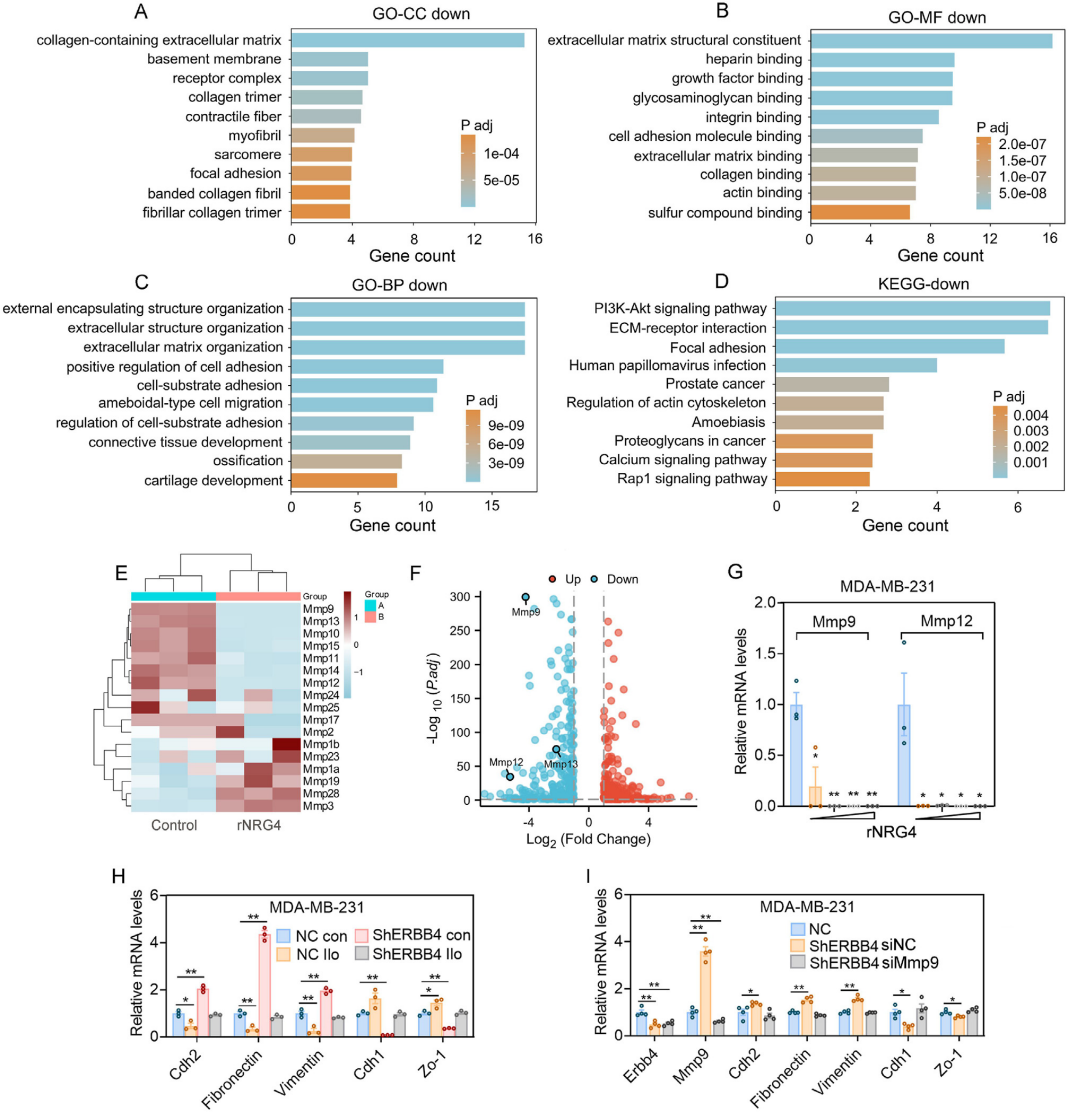
NRG4 suppresses breast cancer metastasis by inhibiting the transcription of MMPs. **(A**–**D)** RNA sequencing data from 4T1 cells treated with rNRG4 revealed a significant decrease in the extracellular matrix composition. **(E)** The heatmap showing commonly dysregulated MMP genes following rNRG4 treatment in 4T1 cells. **(F)** Volcano plot of the differentially expressed genes and labeled MMPs. **(G)** qPCR analysis of the dose-dependent impact of rNRG4 on MMP9 and MMP12 expression. **(H)** qPCR analysis of the effect of the general MMP inhibitor ilomastat on EMT induced by ERBB4 deletion. **(I)** qPCR analysis of the effect of siMMP9 on ERBB4 deletion-induced EMT. The data were presented as mean ± standard deviation. ∗*p* < 0.05 and ∗∗*p* < 0.01.

Subsequently, to further elucidate the downstream signaling pathway of NRG4 in breast cancer cells, we analyzed the RNA sequencing datasets from rNRG4-treated breast cancer cells (Fig. 6A–D; Fig. S6A, B). Our comprehensive analysis of the datasets indicated that rNRG4 suppressed the transcription of MMPs (Fig. 6E and F). Further examination of the expression levels of Mmp9 and Mmp12 demonstrated that both rNRG4 and ERBB4 could inhibit the transcription of MMP9 and MMP12 (Fig. 6G; Fig. S6C, D). Moreover, the general MMP inhibitor ilomastat and shMMP9 both demonstrated suppression of the effect of shERBB4 on EMT, suggesting that MMPs are downstream of ERBB4 (Fig. 6H and I). In addition, the binding of Tead1 and Yap1 was inhibited by rNRG4 treatment (Fig. 5). We also found that both YAP1 and TEAD1 resulted in a slight enhancement of the promoter activity of Mmp9 and Mmp12. Nevertheless, the most significant increase in promoter activity for Mmp9 and Mmp12 was observed when both factors were present simultaneously (Fig. S6E, F). These results collectively indicate the importance of the ERBB4-YAP1-MMPs signaling pathway in regulating the EMT and migration of breast cancer cells.

### rNRG4 suppresses breast cancer organoid progression

**Figure 7:**
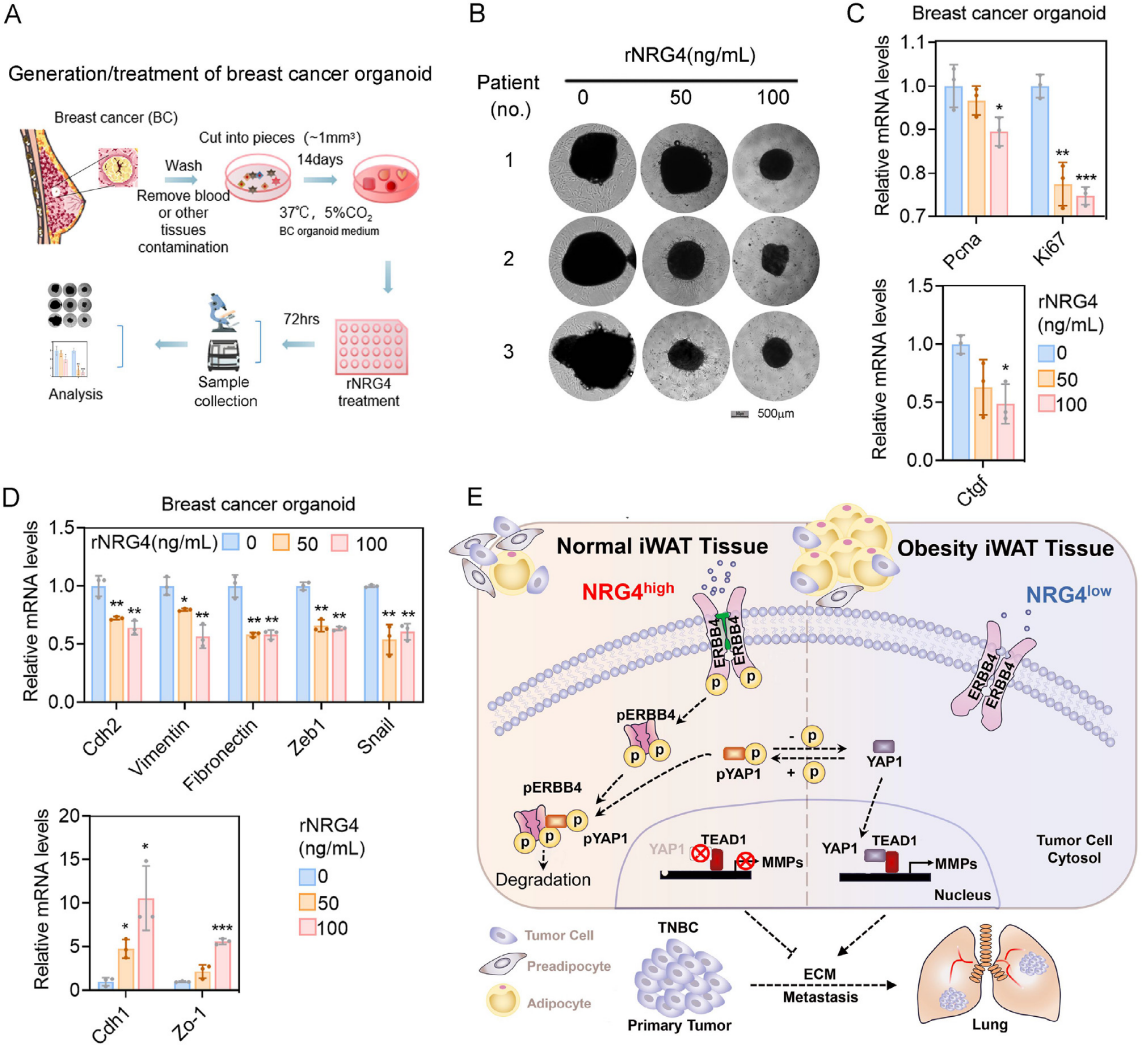
rNRG4 suppresses breast cancer organoid progression. **(A)** Establishment of reproducible breast cancer organoid model. The tumor tissue obtained from the patient was washed and cut into pieces of less than 1 mm in diameter using dissecting scissors. The minced tissue was then resuspended in culture medium. **(B)** Morphology of P0 breast cancer organoids (BCO) treated with 50 or 100 ng/mL rNRG4 for 72 h. **(C)** qPCR analysis of the proliferation (Pcna, Ki67) and angiogenically related genes in BCO treated with 50 or 100 ng/mL rNRG4. **(D)** qPCR analysis of the mesenchymal (Cdh2, fibronectin, vimentin, Zeb1, Snail) and epithelial (Cdh1, Zo-1) related genes in BCO treated with 50 or 100 ng/mL rNRG4. **(E)** The schematic outlines that NRG4 from normal iWAT activates ERBB4 on cancer cells, leading to pERBB4 cleavage and complex formation with pYAP1, preventing YAP1 nuclear translocation and inhibiting tumor metastasis. The data were presented as mean ± standard deviation. ∗*p* < 0.05, ∗∗*p* < 0.01, and ∗∗∗*p* < 0.001.

For clinical relevance, we obtained breast cancer tissue from patients to establish breast cancer organoids (BCO) (Fig. 7A), a promising model for personalized cancer treatment. Consistent with our previous observation, rNRG4 inhibits breast cancer organoid proliferation and the expression of angiogenesis-related genes (Fig. 7B and C) and cell migration-related genes (Fig. 7D). This study demonstrated that NRG4 secreted by adipose tissue activated ERBB4 on breast cancer cells, inducing its phosphorylation. The pERBB4 is subsequently cleaved into a shorter form, which interacts with pYAP1 to inhibit YAP1 nuclear translocation. This process suppresses the transcription of genes associated with extracellular matrix proteins, thereby inhibiting tumor metastasis in breast cancer cells. Conversely, reduced NRG4 levels in the iWAT of obese individuals lead to decreased pERBB4 levels in breast cancer cells. As a result, cytoplasmic pYAP1 is converted to active YAP1, which translocates into the nucleus and drives TEAD1-mediated transcription of Mmp9 and Mmp12, thereby promoting EMT and cell migration. These alterations disrupt the protective effects mediated by NRG4, potentially accelerating tumor progression and metastasis (Fig. 7E).

## Discussion

In this study, we explored the impact of obesity on breast cancer progression using the MMTV-PyMT spontaneous breast cancer model and an *in situ* tumor model (Fig. 1).^34^ Our findings reveal that even mild obesity, induced by an 8-week HFD, significantly promotes lung metastasis of breast cancer, underscoring the sensitivity of tumor progression to metabolic alterations associated with obesity (Fig. 1). These observations align with prior studies demonstrating the complex interplay between adipose tissue and tumors, mediated by diverse cell types, signaling molecules, and pathways.^35^^,^^36^ While the precise signaling mechanisms remain elusive, our study identifies NRG4, secreted by preadipocytes, as a key regulator inhibiting breast cancer metastasis.

Adipocytes, the predominant cell type in adipose tissue, not only store energy but also function as endocrine cells, secreting factors that modulate tumor biology.^35^^,^^36^ While previous research has highlighted the utilization of adipocyte-derived lipids by tumor cells, the role of adipocyte-secreted proteins in regulating cancer cell migration has been less defined. Our study demonstrates that proteins secreted by preadipocytes from NCD-fed mice markedly inhibit breast cancer metastasis, with NRG4 emerging as a critical factor with anti-metastatic properties. Reanalysis of single-cell RNA sequencing data shows that NRG4 is highly expressed in normal iWAT fat cells but significantly down-regulated in obesity. The mechanism underlying reduced NRG4 levels in obesity may involve adipocyte dysfunction, a pro-inflammatory microenvironment, adipose tissue remodeling, and regulation by systemic factors. Specifically, obesity is associated with adipocyte hypertrophy and metabolic dysfunction,^37^ which can impair the ability of mature adipocytes to produce and secrete NRG4. Furthermore, obesity induces a pro-inflammatory state in adipose tissue, characterized by increased macrophage infiltration and elevated levels of inflammatory cytokines such as TNF-α and IL-6.^38^ These inflammatory signals may suppress NRG4 expression, as inflammation is known to down-regulate key adipogenic genes. In addition, the depletion of preadipocytes in obese iWAT, possibly due to impaired adipogenesis or exhaustion of progenitor pools, may contribute to reduced NRG4 levels. Although we did not directly assess preadipocyte numbers in our study, previous research, such as that by Rodeheffer et al, has demonstrated that obesity reduces the preadipocyte pool in adipose tissue, supporting our hypothesis.^39^ Lean iWAT is likely to contain a higher proportion of preadipocytes compared with obese iWAT, which could explain the observed differences in NRG4 levels. We propose that the reduction in NRG4 levels observed in obesity results from a combination of factors, including adipocyte dysfunction, inflammation, altered preadipocyte populations, and systemic metabolic changes. These speculative mechanisms provide a conceptual framework for understanding the link between obesity and reduced NRG4 levels and underscore the need for further studies to elucidate these pathways. Notably, NRG4 levels in adipose tissue correlated inversely with tumor invasion and metastasis, highlighting its paracrine regulatory role in suppressing breast cancer progression.

NRG4 predominantly signals through ERBB4, a receptor implicated in development, tissue homeostasis, and tumor suppression.^40^, ^41^, ^42^ ERBB4 homodimers are thought to act as tumor suppressors, while heterodimers with EGFR or ERBB2 demonstrate oncogenic activity.^40^ This dual role has been observed across various cancers, including bladder, liver, prostate, and breast cancer. Additionally, ERBB4 mutations may serve as biomarkers for cancer staging and therapy. The role of ERBB4 in tumor progression depends on signaling specificity, providing a framework for understanding its involvement in cancer diagnosis, prognosis, and treatment.^39^^,^^40^ This study uncovers a novel tumor-suppressive mechanism of ERBB4. Our results showed that NRG4 up-regulated ERBB4 expression, enhanced its phosphorylation and cleavage, and inhibited EMT and cell migration. Mechanistic studies have revealed that NRG4-stimulated ERBB4 signaling prevents the nuclear translocation of YAP1, a key transcriptional regulator of metastasis-associated genes.^43^, ^44^, ^45^ The interaction between pERBB4 and pYAP1 was confirmed, suggesting a novel mechanism by which NRG4 modulates Hippo pathway activity to suppress metastasis.

To further elucidate downstream signaling, RNA sequencing of breast cancer cells treated with recombinant NRG4 (rNRG4) identified the suppression of MMPs, particularly MMP9 and MMP12, which are critical for extracellular matrix remodeling and tumor invasion. While our results (Fig. 6E) indicate that rNRG4 generally down-regulates several MMP family proteins, including MMP2, MMP9, and MMP12, which suggests a role in inhibiting metastasis, we also observed an up-regulation of MMP3 in response to rNRG4 treatment. This discrepancy may reflect context-dependent regulation, as different MMPs can be involved in various stages of tumor progression or influenced by the specific tumor microenvironment.^46^ Notably, MMP3 has been implicated in both tumor invasion and tissue remodeling, and its up-regulation may indicate a compensatory or differential response in certain contexts.^47^ Thus, while rNRG4 appears to suppress metastasis through the down-regulation of most MMPs, the up-regulation of MMP3 warrants further investigation to better understand the specific mechanisms by which NRG4 modulates MMP expression and its broader implications in breast cancer metastasis. Functional assays validated that NRG4-ERBB4 signaling inhibited the transcription of these MMPs by preventing YAP1-TEAD1 complex formation. Disruption of this pathway effectively impeded EMT and metastasis, providing mechanistic insights into how NRG4 regulates the tumor microenvironment. Aberrant YAP activity has been shown to promote breast cancer and melanoma metastasis in a manner highly dependent on the TEAD interaction domain, which is consistent with our conclusion.^48^

Despite these advances, some limitations continue to exist. While our findings strongly support the role of NRG4 in inhibiting breast cancer metastasis via ERBB4-YAP1 signaling, the exact structural interactions and post-translational modifications of ERBB4 and YAP1 warrant further investigation. Additionally, while clinical data and patient-derived tumor samples suggest that NRG4 and ERBB4 have prognostic significance, the translation of these findings into therapeutic strategies requires comprehensive validation in larger cohorts and preclinical models.

In conclusion, this study identifies NRG4 as a critical adipocyte-derived factor that inhibits breast cancer metastasis, particularly under normal adipose tissue conditions. NRG4 achieves this by modulating the ERBB4-YAP1 axis, suppressing MMP transcription, and impeding EMT. These findings provide novel insights into the metabolic and molecular mechanisms linking obesity and breast cancer, highlighting NRG4 as a promising therapeutic target for obesity-associated cancer progression.

## CRediT authorship contribution statement

**Saijun Wang:** Software, Data curation. **Mingwei Guo:** Validation, Software, Methodology, Investigation, Data curation. **Lingyun Xu:** Software, Formal analysis, Data curation. **Jiaming Xue:** Formal analysis, Data curation. **Shuai Chen:** Validation, Formal analysis. **Ke Xu:** Validation, Formal analysis. **Yan Zhou:** Writing – review & editing. **Aihua Gu:** Resources, Methodology. **Wei Gao:** Resources, Methodology. **Jianwei Zhou:** Writing – review & editing. **Yi Zhang:** Project administration, Conceptualization. **Liming Tang:** Project administration, Funding acquisition, Conceptualization. **Dongmei Wang:** Writing – review & editing, Writing – original draft, Validation, Supervision, Software, Resources, Project administration, Methodology, Investigation, Funding acquisition, Formal analysis, Data curation, Conceptualization.

## Data availability

All data generated or analyzed during this study are included in the article and Supplementary Files, or are available from the corresponding author upon reasonable request.

## Funding

This work was supported by grants from the 10.13039/501100001809National Natural Science Foundation of China (No. 82473051, 82273232), 10.13039/501100004608Natural Science Foundation of Jiangsu Province, China (No. BK20241775), Changzhou Medical Center of Nanjing Medical University Program (Jiangsu, China) (No. CZKY102RC202301, CMC2024PY06, CMCM202403), and Changzhou Sci&Tech Program (Jiangsu, China) (No. LCQYBS202309, LC2024LCQYBS202309, 2024CZBJ020, 2022CZLJ017, LC20242022CZLJ017, QY202306).

## Conflict of interests

The authors declared no conflict of interests.
