# Supplementary material for: NRG4 suppresses breast cancer metastasis via ERBB4-YAP1-mediated down-regulation of MMPs
Source: Genes Dis. 2025 May 16;13(3):101691. doi: 10.1016/j.gendis.2025.101691 (PMC12914539; doi:10.1016/j.gendis.2025.101691)
Supplement: Multimedia component 1 [file mmc1.docx]

**S1 Table. Primers used for plasmid constructions and real-time PCR.**

| **Gene names** | **Forward 5'-3'** | **Reverse 5'-3'** |
| --- | --- | --- |
| hERBB4  (NM_005235.3) | GCAGATGCTACGGACCTTACG | GACACTGAGTAACACATGCTC |
| hNRG  (NM_138573.4) | ATGCCAACAGATCACGAAGAGCC | GTGTTGTTCATGACTGTGGTGG |
| hGapdh (NM_001256799) | CCACTGCCAACGTGTCAGTGGT | CCCAGCAGTGAGGGTCTCTCTCT |
| hCdh1 (NM_001317184) | GGCACAGATGGTGTGATTACAGTC | AGTCTCTCTTCTGTCTTCTGAGGCCA |
| hCdh2 (NM_001308176) | ATGCTGACGATCCCAATGC | GCCTTCCATGTCTGTAGCTTGA |
| hVimentin (NM_003380.5) | GACGCCATCAACACCGAGTT | CTTTGTCGTTGGTTAGCTGGT |
| hFibronectin  (NM_212482.3) | CGGTGGCTGTCAGTCAAAG | AAACCTCGGCTTCCTCCATAA |
| hZo-1 (NM_003257.4) | CAACATACAGTGACGCTTCACA | CACTATTGACGTTTCCCCACTC |
| mGapdh (NM_001289726) | AGGTCGGTGTGAACGGATTTG | GGCCTCACCCCATTTGATGT |
| mCdh1  (NM_009864.3) | CAGGTCTCCTCATGGCTTTGC | CTTCCGAAAAGAAGGCTGTCC |
| mCdh2  (NM_007664.5) | AGCGCAGTCTTACCGAAGG | TCGCTGCTTTCATACTGAACTTT |
| mVimentin  (NM_011701.4) | CGTCCACACGCACCTACAG | GGGGGATGAGGAATAGAGGCT |
| mFibronectin  (NM_010233.2) | ATGTGGACCCCTCCTGATAGT | GCCCAGTGATTTCAGCAAAGG |
| mZo-1  (NM_009386.2) | GCCGCTAAGAGCACAGCAA | TCCCCACTCTGAAAATGAGGA |
| mErbb4  ( NM_010154.2) | GTGCTATGGACCCTACGTTAGT | TCATTGAAGTTCATGCAGGCAA |
| mNrg4  (NM_032002) | CACGCTGCGAAGAGGTTTTTC | CGCGATGGTAAGAGTGAGGA |
| mCtgf  (NM_010217) | GGGCCTCTTCTGCGATTTC | ATCCAGGCAAGTGCATTGGTA |
| mVisfatin  (NM_021524.2 ) | GCAGAAGCCGAGTTCAACATC | TTTTCACGGCATTCAAAGTAGGA |
| mAdiponectin  (NM_009605.5 ) | TGTTCCTCTTAATCCTGCCCA | CCAACCTGCACAAGTTCCCTT |
| mTNF-a  (NM_013693.3 ) | CCCTCACACTCAGATCATCTTCT | GCTACGACGTGGGCTACAG |
| mIL-6  (NM_031168) | TAGTCCTTCCTACCCCAATTTCC | TTGGTCCTTAGCCACTCCTTC |
| mMmp12  (NM_008605) | GAGTCCAGCCACCAACATTAC | GCGAAGTGGGTCAAAGACAG |
| mMmp9  (NM_013599) | CTGGACAGCCAGACACTAAAG | CTCGCGGCAAGTCTTCAGAG |
| mNcald  (NM_001170868) | CAGAGCACGAGATCCAGGAGT | TTTGGAAGCATCCCCGTAAGG |
| hMmp12  (NM_002426) | CATGAACCGTGAGGATGTTGA | GCATGGGCTAGGATTCCACC |
| hMmp9  (NM_004994) | GGGACGCAGACATCGTCATC | TCGTCATCGTCGAAATGGGC |
| mTEAD1  (NM_001166584) | AAGCTGAAGGTAACAAGCATGG | GCTGACGTAGGCTCAAACCC |
| mTEAD2  (NM_011565) | GAAGACGAGAACGCGAAAGC | GATGAGCTGTGCCGAAGACA |
| mTEAD3  (NM_001098226) | CAACCAGCACAATAGCGTCCA | CTGAAAGCTCTGCTCGATGTC |
| mTEAD4  (NM_011567) | CAACCTGGAACATCCCACGAT | GAAAGCCGAGAACTCCAACAT |
| hTEAD1  (NM_021961) | ATGCCAACCATTCTTACAGTGAC | ACAGTTCCTTTAAGCCACCTTTC |
| hTEAD2 (NM_001256660) | GACGGCAGATTTGTGTACCG | GAGACCTCGAAGACATAGGCG |
| hTEAD3 (NM_003214) | TGGACCCTCTCAGGACATCAA | CCAGGGGCTCATAACTGCTG |
| hTEAD4 (NM_201441) | GGACACTACTCTTACCGCATCC | TCAAAGACATAGGCAATGCACA |
| PyMT-MMTV genotyping | GGAAGCAAGTACTTCACAAGGG | GGAAAGTCACTAGGAGCAGGG |
|  | CAAATGTTGCTTGTCTGGTG | GTCAGTCGAGTGCACAGTTT |
| hERBB4-sh1 | Target | GCGCAGGAAACATCTATATTA |
| hERBB4-sh2 | Target | GCCACCAAACATGACTGACTT |
| mERBB4-sh1 | Target | GTACCGAGCCTTGCGCAAATA |
| mERBB4-sh2 | Target | TGTAAGCCTTGCACCGATATT |
| hMmp9-si-1# | sense（5'-3'） | CACGCACGACGUCUUCCAGUAdTdT |
|  | antisense（5'-3'） | UACUGGAAGACGUCGUGCGUGdTdT |
| hMmp9-si-2# | sense（5'-3'） | GCAUAAGGACGACGUGAAUdTdT |
|  | antisense（5'-3'） | AUUCACGUCGUCCUUAUGCdTdT |
| hMmp9 promoter | TAATTGGGGCTGGAGAT | CCTGCCAAAAGACCATGAT |
| hMmp12 promoter | CAGACCGTCCCCATACAATCAGGAGGCA | CCTACATCCTAGATTGTCTAGCGTTG |
