# Supplementary material for: NRG4 suppresses breast cancer metastasis via ERBB4-YAP1-mediated down-regulation of MMPs
Source: Genes Dis. 2025 May 16;13(3):101691. doi: 10.1016/j.gendis.2025.101691 (PMC12914539; doi:10.1016/j.gendis.2025.101691)
Supplement: Multimedia component 2 [file mmc2.docx]

**S2 Table. Antibodies and drugs.**

| **Name** | **SOURCE** | **Catalog Number** |
| --- | --- | --- |
| pERBB4(Y1284) antibody | Cell signaling technology | 4757 |
| ERBB4 antibody | Proteintech | 22387-1-AP |
| ERBB4 antibody | Santa Cruz | sc-71071 |
| pERBB3(Y1289) antibody | Cell signaling technology | 4791 |
| Phospho-EGF Receptor (Tyr1086) Antibody | Cell signaling technology | 2220 |
| p-YAP1(S127) antibody | Abways | CY5743 |
| YAP1 antibody | Proteintech | 13584-1-AP |
| NRG4 antibody | Affinity | DF7046 |
| E-cadherin antibody | BD biosciences | 610182 |
| N-cadherin antibody | BD biosciences | 610921 |
| Vimentin antibody | Abclonal | A19607 |
| Fibronectin antibody | Santa Cruz | sc-8422 |
| β-Tubulin antibody | Beyotime | AF2835 |
| Normal mouse IgG | Santa Cruz | sc-2025 |
| MMP9 antibody (2C3) | Santa cruz | sc-21733 |
| MMP12 antibody (G-2) | Proteintech | 22989-1-AP |
| TEAD1 antibody | Abways | CY9103 |
| Multi-rAB HRP Goat-anti-Mouse | Proteintech | RGAM001 |
| Multi-rAB HRP Goat-anti-Rabbit | Proteintech | RGAR001 |
| LY411575 | Selleck | S2714 |
| Ilomastat (GM6001) | Selleck | S7157 |
