# Supplementary material for: NRG4 suppresses breast cancer metastasis via ERBB4-YAP1-mediated down-regulation of MMPs
Source: Genes Dis. 2025 May 16;13(3):101691. doi: 10.1016/j.gendis.2025.101691 (PMC12914539; doi:10.1016/j.gendis.2025.101691)
Supplement: Multimedia component 3 [file mmc3.docx]

Table S3 Protein list of ERBB4-binding partners in 4T1 cells identified through LC-MS analysis

| **PG.Genes** | **IP_4T1C.** | **IP_4T1N.** | **Log2-fold change** |
| --- | --- | --- | --- |
| Krt76 | 4579788 | 3.74E+07 | 3.029685548 |
| Ppp1r12b | 484569.8 | 2013467 | 2.054905428 |
| Atp6v1b2 | 67030.4 | 278521.9 | 2.054903322 |
| Marcksl1 | 119166.2 | 458558.1 | 1.944129446 |
| Iqgap1 | 163516.8 | 556692.6 | 1.767442038 |
| Mcm2 | 147144.8 | 499115.8 | 1.762138015 |
| Gnb1 | 158737.7 | 521354.9 | 1.71562098 |
| Calr | 188985 | 587650.2 | 1.636685913 |
| Dynll1 | 353808.2 | 1083616 | 1.614814211 |
| Fgb | 81546.84 | 244918.7 | 1.586602053 |
| Krt2 | 4972551 | 1.43E+07 | 1.523957074 |
| Ganab | 206448.2 | 560163.6 | 1.440068399 |
| Psmc2 | 152781.9 | 412476.6 | 1.432838639 |
| Srm | 76837.59 | 205008 | 1.415796034 |
| Eif2s1 | 345205.6 | 852941.9 | 1.304991604 |
| Psmd12 | 457304.9 | 1057841 | 1.209894515 |
| Epb41l2 | 177731.2 | 410639.1 | 1.208174041 |
| Pabpn1 | 136747.8 | 313992.6 | 1.199212936 |
| Rab1A | 453466.3 | 1031459 | 1.185619232 |
| Hadhb | 166876.9 | 378260.6 | 1.180596249 |
| Hspe1 | 3139949 | 6900815 | 1.13602563 |
| Uqcrc1 | 161550.4 | 352566.5 | 1.125911077 |
| Rpl24 | 930975.2 | 2029489 | 1.124301879 |
| Rcc2 | 675120.4 | 1449718 | 1.102555575 |
| Armcx5 | 171078.4 | 364975.5 | 1.093142003 |
| Txnrd1 | 214901.5 | 458132.3 | 1.092088729 |
| Akap2 | 561342.4 | 1185361 | 1.078373557 |
| Galk1 | 293795.3 | 604366.3 | 1.0406119 |
| Rps15a | 995000 | 2033365 | 1.031100779 |
| Cep162 | 603150 | 1228494 | 1.02630207 |
| Gdi2 | 436635.2 | 884505.8 | 1.018443163 |
| Eif2s3x | 269315.9 | 544443.4 | 1.015482668 |
| Actb | 3.86E+07 | 7.79E+07 | 1.013022481 |
| H3-5 | 2601736 | 1290602 | -1.011430412 |
| Calu | 1397763 | 693162.5 | -1.011854251 |
| Eef1g | 8915482 | 4420831 | -1.011995213 |
| Rplp2 | 1130713 | 559868.4 | -1.014073129 |
| Ywhag | 2327882 | 1151729 | -1.015216637 |
| Srrm2 | 610109.3 | 301802.1 | -1.015464878 |
| Serpinc1 | 452089.1 | 223305.1 | -1.017590933 |
| Tcp1 | 4741975 | 2320228 | -1.031221476 |
| Sh3bgrl | 554143.2 | 270066.3 | -1.036945216 |
| Cars1 | 323357.6 | 157477.2 | -1.037987553 |
| Yap1 | 1237585 | 599130.8 | -1.04658471 |
| RTRAF | 421956.8 | 202441.6 | -1.059589521 |
| Pfkl | 1091846 | 523585.6 | -1.06027206 |
| Bag3 | 463260.1 | 221862.3 | -1.062157892 |
| Iws1 | 3204226 | 1522512 | -1.073522308 |
| Mcm6 | 345578.8 | 163567.6 | -1.079127713 |
| Tmod3 | 8755169 | 4128412 | -1.084548074 |
| Hnrnpk | 1877685 | 884664.4 | -1.085752884 |
| Rars1 | 331306.4 | 155628 | -1.090064425 |
| Myo5a | 859191.8 | 400051.3 | -1.102795211 |
| Krt19 | 315391.7 | 146459.2 | -1.106645873 |
| Hspa4l | 3432139 | 1570285 | -1.128081557 |
| NaN | 1759805 | 804687.4 | -1.128915228 |
| Hint1 | 1048054 | 477233.2 | -1.134946736 |
| Ssr4 | 344912.8 | 156822.8 | -1.137096346 |
| Serpinh1 | 410749 | 185045.5 | -1.150377011 |
| Suclg1 | 640015.3 | 287069.6 | -1.156705834 |
| Pgam5 | 569672.6 | 252542.3 | -1.173607963 |
| Tpm1 | 5.45E+07 | 2.41E+07 | -1.177223083 |
| Gtf2i | 1005187 | 443115.6 | -1.181708895 |
| Hmga2 | 1000316 | 440964.7 | -1.181720744 |
| Septin2 | 1031997 | 446360.4 | -1.209157831 |
| Coro1c | 8239332 | 3555577 | -1.212443677 |
| Tfrc | 402063.8 | 173299.8 | -1.214154459 |
| Thop1 | 221519.3 | 94876.35 | -1.223311986 |
| Rpl11 | 2332086 | 998281.3 | -1.224102685 |
| P4hb | 3534487 | 1507341 | -1.229495006 |
| Hmgb2 | 1859661 | 791693.3 | -1.232026107 |
| Ppp2r1a | 1016177 | 431981.1 | -1.234111618 |
| Ube2n | 520885 | 218449.3 | -1.253666409 |
| Nucks1 | 469806.2 | 196179.6 | -1.259890724 |
| Rps23 | 3332928 | 1375392 | -1.276947293 |
| Tubb6 | 1043855 | 430307.1 | -1.278482774 |
| Ssbp2 | 224959.1 | 91982.34 | -1.290233923 |
| Pcbp1 | 1884228 | 764163.8 | -1.302019727 |
| Ldb1 | 3059832 | 1233733 | -1.310422238 |
| Pcm1 | 721333.6 | 289615.3 | -1.316528807 |
| Obi1 | 990257.1 | 396859.4 | -1.319175163 |
| Anln | 393633.2 | 151962.6 | -1.373135605 |
| Eif2s2 | 1355913 | 513974.2 | -1.399496766 |
| Eif4g1 | 2033473 | 770253.2 | -1.400541159 |
| Golga3 | 1.86E+07 | 7033039 | -1.403082499 |
| Rpl14 | 2412538 | 912179.5 | -1.403162013 |
| Vcp | 3.45E+07 | 1.29E+07 | -1.419225296 |
| Qars1 | 574330.6 | 211567.6 | -1.440762723 |
| Capzb | 4015873 | 1477191 | -1.442857268 |
| Rack1 | 3135662 | 1146785 | -1.451175117 |
| Pcnp | 412310.3 | 150652.9 | -1.452502057 |
| Hspa5 | 1.32E+07 | 4727450 | -1.481403825 |
| Vasp | 1866893 | 662720.2 | -1.494167443 |
| Tpi1 | 6101567 | 2142380 | -1.509965404 |
| Pdcd6ip | 1334828 | 462637.2 | -1.528700674 |
| Rpl19 | 4091578 | 1411942 | -1.534976529 |
| Cyfip1 | 369053.7 | 126950 | -1.539570359 |
| Capg | 909756 | 312862.5 | -1.539950915 |
| Rps20 | 910108.5 | 311096.4 | -1.548676849 |
| Rtcb | 1016412 | 342388.9 | -1.569777477 |
| Ppa1 | 254733.4 | 85627.25 | -1.572846237 |
| Chchd3 | 1499203 | 503553.9 | -1.573977629 |
| Cox5b | 888375.9 | 298382.2 | -1.574008782 |
| Dld | 603068.7 | 201336.1 | -1.582716486 |
| Abce1 | 835066.1 | 275399.9 | -1.600362364 |
| Copb2 | 489712.5 | 160472.3 | -1.609610734 |
| Mif | 1469154 | 481095.6 | -1.61059012 |
| Rpl35a | 6783587 | 2217896 | -1.612856621 |
| Etfa | 715744.3 | 233118.9 | -1.618378301 |
| Idh2 | 553674.4 | 180056.2 | -1.62059054 |
| Mapk1 | 605464.9 | 195822.8 | -1.628494576 |
| Rps10 | 3910602 | 1260758 | -1.633099334 |
| Nasp | 500435.5 | 160586.9 | -1.639829927 |
| Pfn1 | 1010013 | 321685.9 | -1.650649255 |
| Rhoa | 622138 | 195334.4 | -1.671288586 |
| G3bp2 | 906163.8 | 280649.9 | -1.691000313 |
| Eif4e | 662026.6 | 201353 | -1.717162218 |
| Rps2 | 6084394 | 1845049 | -1.721454448 |
| Psma1 | 1088224 | 329375.6 | -1.724169962 |
| Sf3b3 | 316271.8 | 94608.84 | -1.741118029 |
| Shmt2 | 3328889 | 969792.9 | -1.779292169 |
| Cct6a | 4108895 | 1176618 | -1.804104452 |
| Ppp1r13l | 3396466 | 969230.8 | -1.809122256 |
| Tra2b | 695723.5 | 198048.6 | -1.81265955 |
| Hadha | 429351.5 | 120102.6 | -1.837891849 |
| Eif3b | 433191.8 | 119993 | -1.852055688 |
| Rpl12 | 1701811 | 468682.4 | -1.860388298 |
| Eif3e | 665283.3 | 182643.5 | -1.864938408 |
| Hnrnpf | 1983523 | 537739.9 | -1.883084699 |
| Cdc37 | 925426.5 | 250543.9 | -1.885054999 |
| Phb1 | 1330477 | 356531.1 | -1.899843738 |
| Ppp1r18 | 2112140 | 551147.6 | -1.938194828 |
| Bub3 | 433744.3 | 113173.6 | -1.938307339 |
| Nedd8 | 241089.9 | 61728.38 | -1.965565378 |
| Lrrfip1 | 1418014 | 359049.4 | -1.98161752 |
| Lars1 | 842136.4 | 208538.9 | -2.013737401 |
| Psma5 | 1148512 | 267877.6 | -2.100120078 |
| Sbno1 | 777878.4 | 177387.5 | -2.132640296 |
| Grpel1 | 1143184 | 248462 | -2.201960503 |
| Capza2 | 1889033 | 410285.8 | -2.202946776 |
| Acat1 | 423467.3 | 89653.42 | -2.239820046 |
| Rps27 | 2166540 | 445357.3 | -2.282357726 |
| Ctnnb1 | 807860.1 | 165612.5 | -2.28629391 |
| Tnip1 | 934636.6 | 185711.9 | -2.331339271 |
| Clip2 | 813063.1 | 161547.4 | -2.331409789 |
| Arpc3 | 8425388 | 1626689 | -2.372804671 |
| Prpf39 | 966527.3 | 166747.4 | -2.535146216 |
| Dars1 | 842208.3 | 143427 | -2.553860458 |
| Eef1d | 4705310 | 748889.9 | -2.651464237 |
| Hnrnpa3 | 1841008 | 291032.1 | -2.661245704 |
| Rps13 | 2408106 | 379348.8 | -2.666302019 |
| Dbi | 1997484 | 311351.7 | -2.681566886 |
| Sec31a | 1738316 | 267007.8 | -2.702736574 |
| Krt17 | 3.39E+07 | 4556249 | -2.895366775 |
| Ahcy | 1352464 | 178599.2 | -2.920792669 |
| Nudt21 | 1.20E+07 | 1494575 | -3.005227205 |
| Ppp1ca | 2851848 | 352127.3 | -3.017728103 |
| Fh | 1031211 | 112271.9 | -3.199270765 |
| Cct7 | 2457820 | 239201 | -3.361083937 |
| S100a11 | 6348460 | 615440.3 | -3.366715846 |
| Spag9 | 3572329 | 279649.1 | -3.675175362 |
| Myl6b | 7.82E+07 | 3365821 | -4.538138249 |
