# Supplementary material for: NRG4 suppresses breast cancer metastasis via ERBB4-YAP1-mediated down-regulation of MMPs
Source: Genes Dis. 2025 May 16;13(3):101691. doi: 10.1016/j.gendis.2025.101691 (PMC12914539; doi:10.1016/j.gendis.2025.101691)
Supplement: Multimedia component 4 [file mmc4.docx]

**Supplementary Figures**

**Figure S1** Breast cancer model in high-fat-diet-fed mice. Immunohistochemistry staining of vimentin in primary tumors and liver tissue from 4T1-bearing mice fed with a normal-chow diet or high-fat diet.

**Figure S2** Co-culture of tissues and breast cancer cells. **(A)** Co-culture of equal amounts of mammary tissue from normal-chow-diet-fed or high-fat-diet-fed mice with breast cancer cells, followed by transwell and quantitative reverse-transcription PCR experiments for assessing cell migration and the expression of key epithelial-mesenchymal-transition-related genes. **(B)** Co-culture of equal amounts of inguinal white adipose tissue (iWAT) from normal-chow-diet-fed or high-fat-diet-fed mice with breast cancer cells, followed by transwell and quantitative reverse-transcription PCR experiments for assessing cell migration and the expression of key epithelial-mesenchymal-transition-related genes. The data were presented as mean ± standard error of the mean. ^*^*P* < 0.05 and ^**^*P* < 0.01.

**Figure S3** Principal component analysis and volcano plot of conditional medium proteinomic data. **(A)** Principal component analysis of liquid chromatography-mass spectrometry data. **(B)** Volcano plot analysis of liquid chromatography-mass spectrometry data. The red color indicates an increase, and the blue color represents a decrease in the proteins of adipocytes compared with preadipocytes.

**Figure S4** NRG4 inhibits breast cancer metastasis. **(A)** Immunohistochemistry staining of vimentin in primary tumors and liver tissue from 4T1-bearing mice treated with rNRG4. **(B)** Weight analysis of primary tumors (left) and lung tissues (middle), and lung metastases (right) of PyMT-MMTV mice (*n* = 6). **(C)** Hematoxylin-eosin staining of lung tissues of PyMT-MMTV mice. **(D**) CCK8 assay assessed the proliferation of 4T1 or MDA-MB-231 cells co-cultured with preadipocytes or adipocytes for 24 h. The data were presented as mean ± standard error of the mean. ^*^*P* < 0.05.

**Figure S5** The expression of ERBB4 in BRCA tissues. **(A)** UALCAN analysis of ERBB4 expression in BRCA across major subclasses (including triple-negative breast cancer types) of the TCGA database. **(B)** Immunohistochemistry experiments revealed significantly lower ERBB4 expression in both the primary tumor and lung tissue of high-fat-diet-fed mice. **(C)** Immunohistochemistry experiments revealed significantly higher ERBB4 expression in both the primary tumor and lung tissue of rNRG4-treated high-fat-diet-fed mice. **(D)** CCK8 assay assessed the proliferation of 4T1 or MDA-MB-231 cells co-cultured with preadipocytes or adipocytes for 24 h. **(E)** Immunofluorescence assays were used to detect the expression and cytoplasmic distribution of pYap1(S127) and ERBB4 in 4T1 cells overexpressing ERBB4. The data were presented as mean ± standard error of the mean. ^*^*P* < 0.05 and ^**^*P* < 0.01.

**Figure S6** Effect of rNRG4 and ERBB4 on the expression of Mmp9 and Mmp12. **(A)** Gene Ontology (GO) and Kyoto Encyclopedia of Genes and Genomes (KEGG) analyses of the up-regulated genes in rNRG4-treated 4T1 cells. **(B)** GO and KEGG analysis of the down-regulated genes in rNRG4-treated 4T1 cells. **(C)** Immunoblotting analysis of Mmp9 and Mmp12 in MDA-MB-231 cells treated with rNRG4. **(D)** Immunoblotting analysis of Mmp9 and Mmp12 in MDA-MB-231 cells overexpressing ERBB4. **(E)** Luciferase activity of the Mmp9 promoter in the presence of Yap1 and/or Tead1 in 293T cells. **(F)** Luciferase activity of the Mmp12 promoter in the presence of Yap1 and Tead1 in 293T cells. The data were presented as mean ± standard error of the mean. ^*^*P* < 0.05 and ^**^*P* < 0.01.
