# Supplementary figures and images for: NRG4 suppresses breast cancer metastasis via ERBB4-YAP1-mediated down-regulation of MMPs
Source: Genes Dis. 2025 May 16;13(3):101691. doi: 10.1016/j.gendis.2025.101691 (PMC12914539; doi:10.1016/j.gendis.2025.101691)

Fig. S1

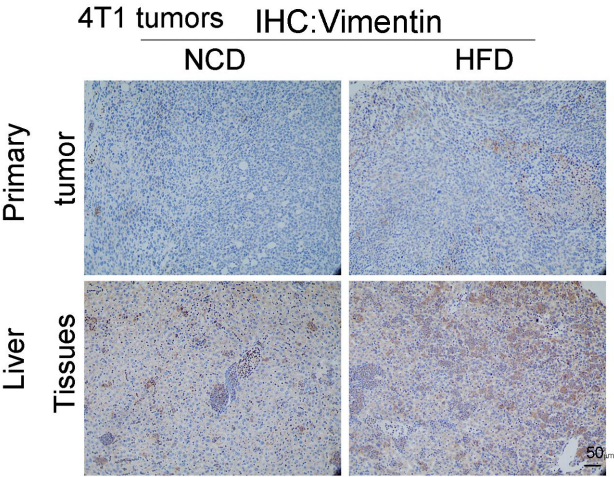

**Fig. S2**

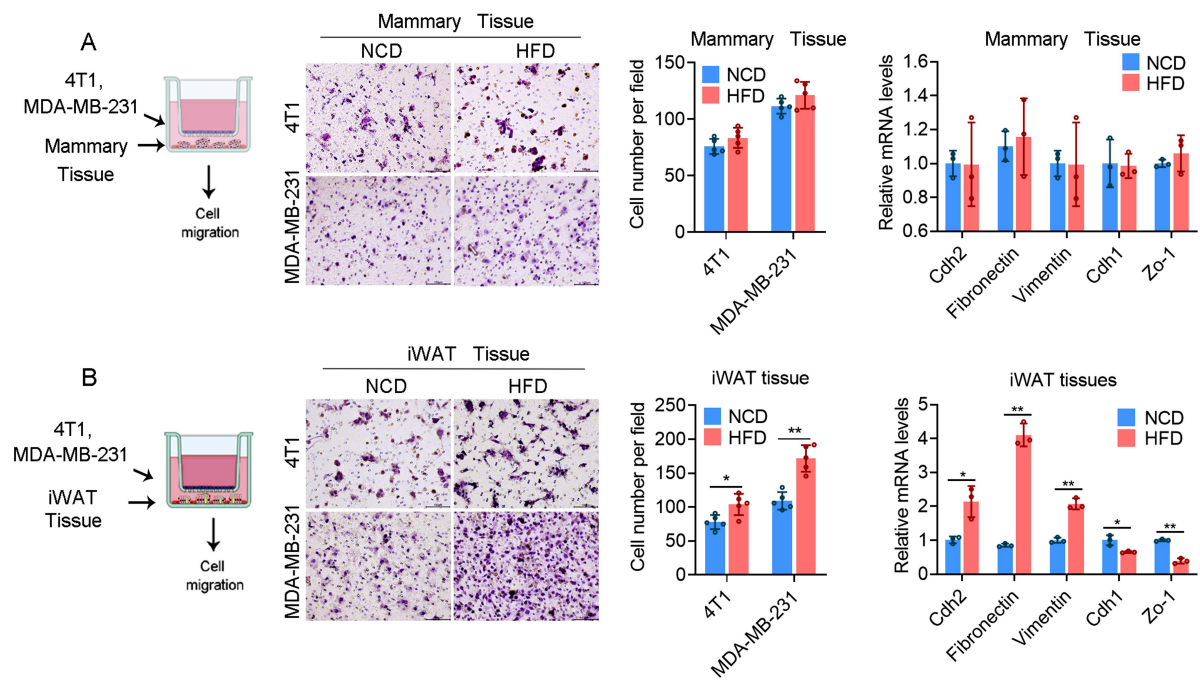

Fig. S3

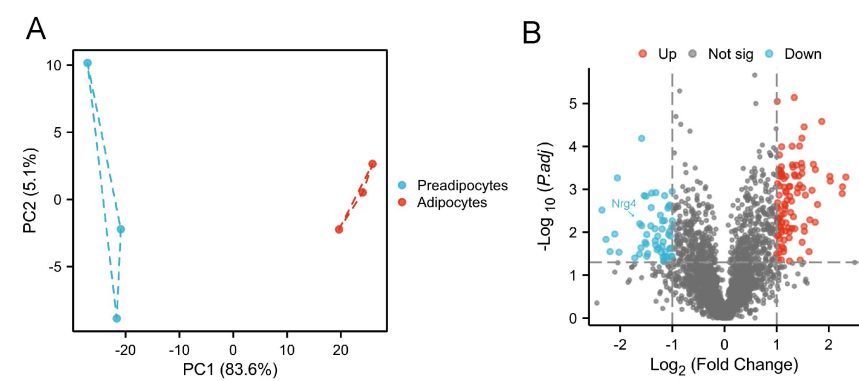

Fig. S4

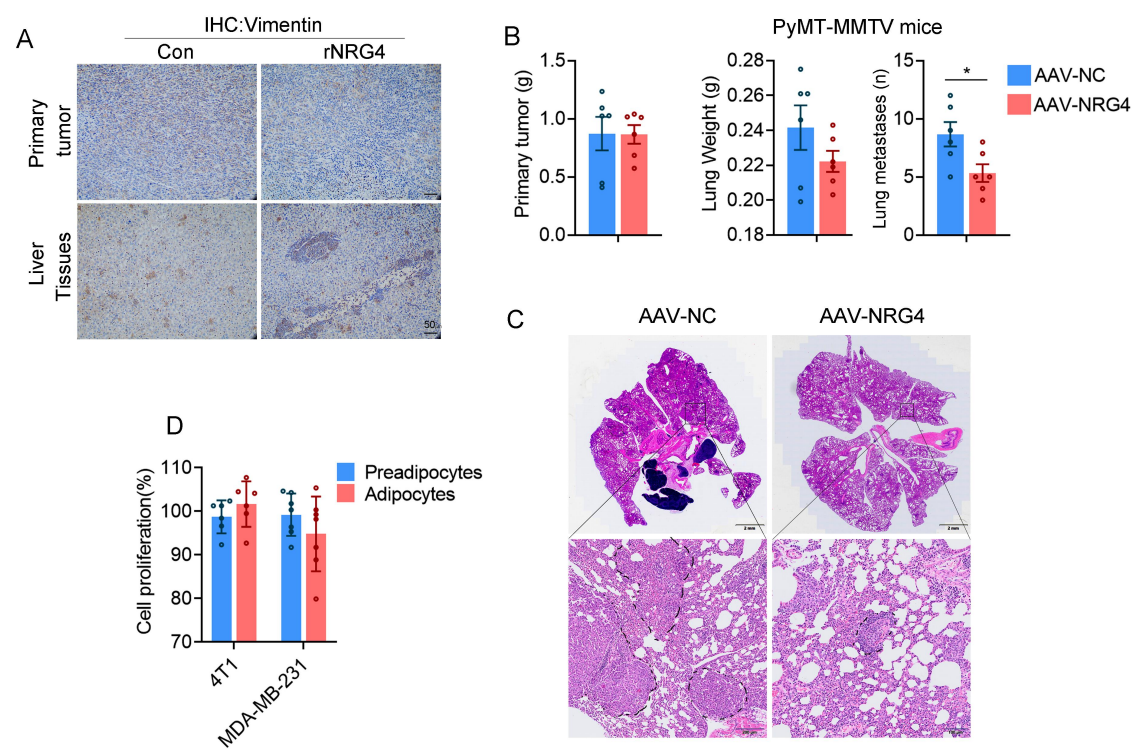

Fig. S5

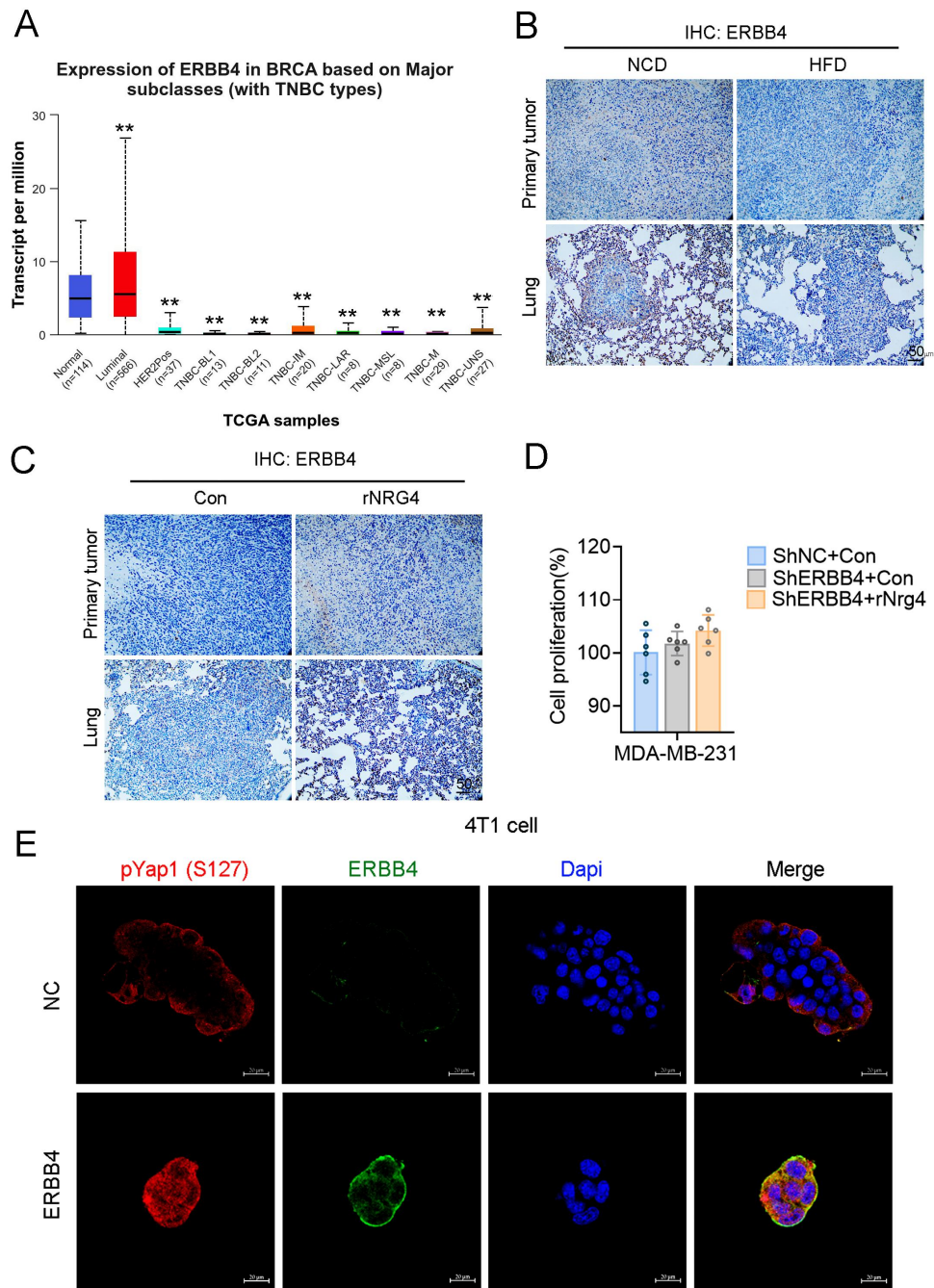

**Fig. S6**

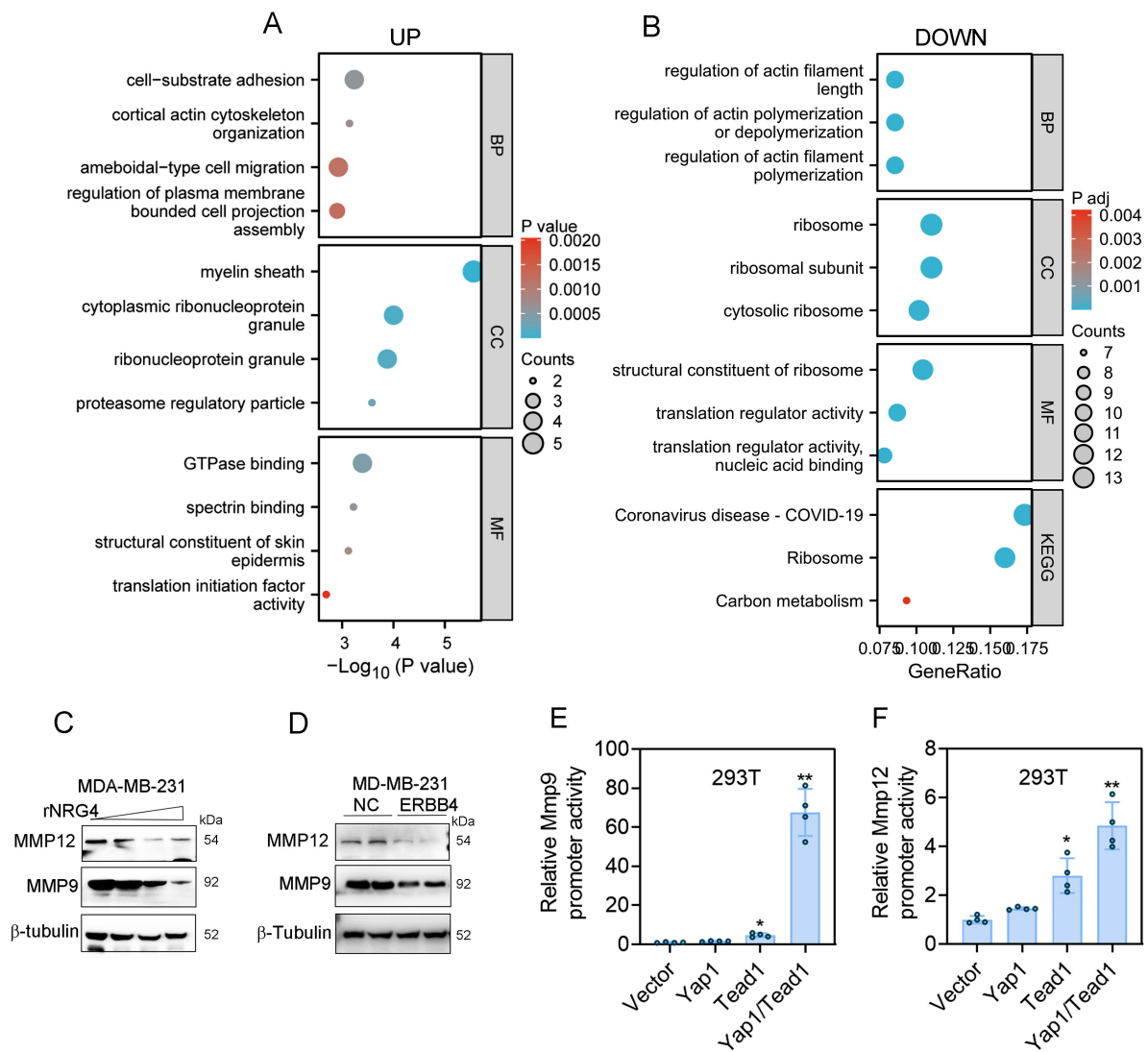

Supplement: Multimedia component 5 [file mmc5.pdf]
